# Improved Transglycosylation by a Xyloglucan-Active α-l-Fucosidase from *Fusarium graminearum*

**DOI:** 10.3390/jof6040295

**Published:** 2020-11-18

**Authors:** Birgitte Zeuner, Marlene Vuillemin, Jesper Holck, Jan Muschiol, Anne S. Meyer

**Affiliations:** Department of Biotechnology and Biomedicine, Technical University of Denmark, 2800 Kgs. Lyngby, Denmark; bzeu@dtu.dk (B.Z.); mavu@dtu.dk (M.V.); jesho@dtu.dk (J.H.); jmus@dtu.dk (J.M.)

**Keywords:** fucosidase, xyloglucan, 2′-fucosyllactose, human milk oligosaccharides, protein engineering, GH29

## Abstract

*Fusarium graminearum* produces an α-l-fucosidase, *Fg*FCO1, which so far appears to be the only known fungal GH29 α-l-fucosidase that catalyzes the release of fucose from fucosylated xyloglucan. In our quest to synthesize bioactive glycans by enzymatic catalysis, we observed that *Fg*FCO1 is able to catalyze a transglycosylation reaction involving transfer of fucose from citrus peel xyloglucan to lactose to produce 2′-fucosyllactose, an important human milk oligosaccharide. In addition to achieving maximal yields, control of the regioselectivity is an important issue in exploiting such a transglycosylation ability successfully for glycan synthesis. In the present study, we aimed to improve the transglycosylation efficiency of *Fg*FCO1 through protein engineering by transferring successful mutations from other GH29 α-l-fucosidases. We investigated several such mutation transfers by structural alignment, and report that transfer of the mutation F34I from *Bi*AfcB originating from *Bifidobacterium longum* subsp. *infantis* to Y32I in *Fg*FCO1 and mutation of D286, near the catalytic acid/base residue in *Fg*FCO1, especially a D286M mutation, have a positive effect on *Fg*FCO1 transfucosylation regioselectivity. We also found that enzymatic depolymerization of the xyloglucan substrate increases substrate accessibility and in turn transglycosylation (i.e., transfucosylation) efficiency. The data include analysis of the active site amino acids and the active site topology of *Fg*FCO1 and show that transfer of point mutations across GH29 subfamilies is a rational strategy for targeted protein engineering of a xyloglucan-active fungal α-l-fucosidase.

## 1. Introduction

In nature, synthesis of glycosidic linkages is catalyzed by glycosyl transferases (GTs), whereas their hydrolysis is catalyzed by glycoside hydrolases (GHs). However, GHs operating via the double-displacement retaining mechanism or via substrate-assisted catalysis (exploiting a 2-acetamido group of the substrate as nucleophile) can also catalyze synthesis of glycosidic bonds through transglycosylation. This happens when the intermediate encounters another acceptor molecule than water [1]. This transglycosylation ability makes GHs attractive to exploit for glycan synthesis. However, since transglycosylation catalyzed by GHs takes place in competition with substrate hydrolysis, native GHs are often inefficient transglycosylation catalysts. Engineering of GHs to improve their transglycosylation efficiency is thus an important strategy to expand the range of glycans that can be produced in vitro [2,3,4]. GHs are often preferred over GTs for in vitro catalysis due to their easier recombinant expression, robustness, and their ability to use naturally abundant substrates (GTs require sugar-1-phosphate derivatives, for fucosyl transferases even including expensive diphospho-nucleotides as reaction substrates) [4].

l-Fucose (Fuc) is present in a variety of glycans in the human body, such as receptors on the cell surface, blood group antigens, and human milk oligosaccharides (HMOs), and is involved in a wide range of biological functions [5]. The use of α-l-fucosidases for transglycosylation, i.e., transfucosylation, therefore often aims at the synthesis of glycans mimicking those found in the human body. So far, much of the transfucosylation focus has been on the synthesis of HMOs [6,7,8,9], which to a large extent resemble the blood group antigen structures. Importantly, GH engineering not only expands the synthetic portfolio of specific glycans, but also provides insight into the structure–function relationships governing the balance between hydrolysis and transglycosylation in general.

In the CAZy database of carbohydrate-active enzymes (www.cazy.org [10]), α-l-fucosidases are currently classified in three different GH families: GH29, GH95, and GH151. GH95 comprises α-l-fucosidases (EC 3.2.1.51) and α-1,2-l-fucosidases (EC 3.2.1.63) with an inverting reaction mechanism. This mechanism renders them unsuited for transglycosylation beyond transformation into fucosynthases [11]. GH151 is a new family comprising only a few, poorly characterized members, but a retaining mechanism has been inferred based on a few reports of transglycosylation activity [9,12]. GH29 comprises α-l-fucosidases (EC 3.2.1.51), α-1,3/4-l-fucosidases (EC 3.2.1.111) and a single α-1,2-l-fucosidase (EC 3.2.1.63), which all employ a retaining double-displacement reaction mechanism. Several GH29 members have been employed for transfucosylation [6,7,8,13,14,15] and for fucosylation by reverse hydrolysis [16]. GH29 has been divided into subfamilies GH29A and GH29B based on sequence homology and the resulting substrate specificity: members of GH29B are regiospecific α-1,3/4-l-fucosidases targeting only α-1,3- or α-1,4-linkages with a branched galactose (Gal) residue, whereas members of GH29A have a more relaxed fucosyl substrate specificity [17]. 

While terminal Fuc plays an important role in the human body [5], it is comparatively rare in plant biomass, but may occur on substituted xyloglucans. Xyloglucan substitution is highly dependent on species, tissue, and developmental stage, but fuco-galactoxyloglucan is common in dicots, i.e., most vegetables and fruit, where Fuc is present in a terminal position α-1,2-linked to Gal [18]. Among the few α-l-fucosidases known to catalyze release of Fuc from xyloglucan, several are inverting GH95 α-l-fucosidases of plant origin [19,20]. Similarly, only GH95 α-l-fucosidases were identified in the xyloglucan utilization loci of several *Bacteroides* sp. and one *Dysgonomonas* sp. [18]. So far, only two GH29 members have been found active on xyloglucan: *Fg*FCO1 and the bacterial Mfuc5 from a soil metagenome [7,9,21]. This emphasizes the ample space for future discoveries of novel xyloglucan-active GH29 α-l-fucosidases in fungi and bacteria.

Previously, the transfucosylation potential of the two xyloglucan-active GH29A α-l-fucosidases *Fg*FCO1 and Mfuc5 was investigated [7]. Transferring Fuc from citrus peel xyloglucan to lactose, *Fg*FCO1 produced 2′-fucosyllactose (Fuc-α-1,2-Gal-β-1,4-Glc; 2′FL), whereas Mfuc5 mainly catalyzed formation of a fucosyllactose isomer different from HMO structures 2′FL and 3-fucosyllactose (Gal-β-1,4-[Fuc-α-1,3-]Glc; 3FL). Using an acceptor-to-donor ratio of 50 (2 mM Fuc bound in xyloglucan and 100 mM lactose), the molar transfucosylation yield on the donor reached merely 14% when employing *Fg*FCO1 for 24 h [7]. Consequently, the current study aims to improve the transglycosylation efficiency and maximize regioselectivity of *Fg*FCO1 through protein engineering. Enzymatic depolymerization of the xyloglucan substrate as a means to increase substrate accessibility and in turn transfucosylation efficiency is also studied.

Setting up a high-throughput screening system for directed evolution is complicated for transglycosylation, which takes place in competition with hydrolysis, but can be achieved if the GH is active on a chromogenic substrate [13,22,23,24]. However, *Fg*FCO1 is not active on *p*NP-α-l-Fuc [7,25]. Instead, we aimed to investigate the potential of transferring mutations reported in previous successful fucosidase engineering studies—recently reviewed elsewhere [26]. Successful transfer of a single point mutation between members of the same GH family to improve transglycosylation has been shown for three different thermostable GH1 β-galactosidases (sequence identity 27–52%) [27]. The division of GH29 into two subfamilies is linked to structural differences in the active site surroundings [17,28], which may complicate direct transfer of successful mutations between the two families. Indeed, in a previous attempt to transfer successful point mutations from GH29A to GH29B, only one out of three of the amino acids in question were found to align well in the three-dimensional structures [6]. Thus, this study aims to provide further knowledge on the transferability of engineering results within a GH family containing subfamilies in order to facilitate future rational design. The strategy includes selection of the best mutations obtained in previous studies followed by careful structural alignment and sequence alignment to *Fg*FCO1. Where good alignments are obtained, the same mutations are introduced in *Fg*FCO1 and tested. Importantly, mutations are transferred from bacterial fucosidases inactive on fucosylated xyloglucan to the fungal xyloglucan-active *Fg*FCO1, which is then tested on xyloglucan. It is hypothesized that the effect of point mutations on transfucosylation efficiency is transferable between the two subfamilies as long as the amino acid in question appears in a similar position in the three-dimensional structures of the wild-type (WT) enzymes. Furthermore, it is expected that *Fg*FCO1 will maintain its transfucosylation activity on xyloglucan after engineering.

## 2. Materials and Methods 

### 2.1. Chemicals

2′-fucosyllactose (2′FL) and 3-fucosyllactose (3FL) were purchased from Elicityl Oligotech (Crolles, France). l-fucose (Fuc), β-lactose, and all other chemicals were purchased from Sigma-Aldrich (Steinheim, Germany). Dried citrus peel was supplied by Dupont Nutrition BioSciences ApS (Brabrand, Denmark) and used for alkaline extraction of the xyloglucan-rich fraction containing 12 mg Fuc per g dry matter as described previously [7]: The xyloglucan was extracted in a solution of 24% (*w*/*v*) KOH with 0.1% (*w*/*v*) NaBH_4_. After neutralization with acetic acid, the solution was first filtered on a 0.22 μm to remove large particles and then diafiltered on a 5 kDa cellulose membrane (Vivaflow 200; Sartorius AG, Göttingen, Germany) to remove salt and other small molecules including smaller plant cell wall fragments. Finally, the extract was freeze-dried and kept dry at room temperature until further use.

### 2.2. Xyloglucanase Treatment of Citrus Peel Xyloglucan

Freeze-dried citrus peel xyloglucan was dissolved in 100 mM acetate buffer (pH 4.6) to a concentration corresponding to 8 mM bound Fuc (approximately 0.1 g/L). To investigate the effect of substrate depolymerization by xyloglucanase treatment, the extracted xyloglucan was treated with a GH5 *Paenibacillus* sp. xyloglucan-specific endo-β-1,4-glucanase (XEG; EC 3.2.1.151; Megazyme, Wicklow, Ireland) using 100 U/g substrate at 40 °C for 1 h. The xyloglucanase was heat-inactivated for 10 min at 99 °C. The untreated, extracted xyloglucan was subjected to the same heat treatment without enzyme addition. Size distributions of the two xyloglucan preparations were determined by size exclusion chromatography (SEC) as described previously [29] using a Shodex SB-806 HQ GPC column (300 mm × 8 mm) equipped with a Shodex SB-G guard column (50 mm × 6 mm; Showa Denko K.K., Tokyo, Japan), an UltiMate iso-3100 SD pump (Dionex, ThermoFisher Scientific, Waltham, MA, USA), and an RI-101 refractive index detector (Showa Denko K.K., Tokyo, Japan). Elution took place with a 100 mM sodium acetate eluent at a flow rate of 0.5 mL/min at 40 °C. Pullulan standards were used as the reference for molecular weight. 

### 2.3. In Silico Methods

Crystal structures of *Fg*FCO1 (PDB 4NI3; closed form), *Bi*AfcB (PDB 3UES; closed form in complex with inhibitor), and *Tm*αFuc (PDB 1HL9; closed form in complex with an inhibitor; and PDB 2ZWY; closed form) were used for creating structural alignments in PyMOL Version 2.3.3 (Schrödinger, New York, NY, USA) using the super alignment function due to low sequence identity between the three enzymes (25–31%). Since PDB 1HL9 misses important residues, a model for *Tm*αFuc was created on PDB 1HL9 and PDB 2ZWY. Ligands from *Fg*FCO1 (PDB 4PSR) and *Bi*AfcB (PDB 3UET) were aligned with 4NI3 and 3UES, respectively, by the align function. Homology models of *Fg*FCO1 variants were made in YASARA Structure 16.12.9 (YASARA Biosciences GmbH, Vienna, Austria) by swapping the target amino acid residue to the desired mutation in the *Fg*FCO1 crystal structure with ligand (PDB: 4PSR) using the in-built function of the program. The resulting structure was refined using the YASARA macro md_refine (standard settings), which performs a 500 ps simulation of a homology model using the protocol described previously [30]. The snapshot with the highest quality score from each refinement run was structurally aligned with the WT using the align function in PyMOL and visually inspected for differences. Multiple sequence alignments were made with MUSCLE [31] using default settings. The alignment was visualized by ESPript 3.0 [32] using PDB 4NI3 (*Fg*FCO1) as template for the structural alignment. For consistency in *Fg*FCO1 across publications, the numbering from the crystal structure [25] was used, i.e., the 24-amino acid signal peptide given in the GenBank sequence (AFR68935.1) was omitted from the numbering.

### 2.4. Site-Directed Mutagenesis of FgFCO1

All mutants were constructed using CloneAmp^TM^ polymerase (Takara, Kusatsu, Japan), a set of mutagenic primers, and pPICZα/*Fg*FCO1 WT [7] as template or the appropriately mutated template for the construction of double and triple mutants. The different primers used for site-directed mutagenesis are listed in Table S1. The WT plasmid was then digested by DpnI at 37 °C overnight and the PCR products were purified using the Illustra GFX PCR DNA and Gel Band Purification Kit (GE Healthcare Life Sciences, Chicago, IL, USA). Purified PCR products were transformed into *Escherichia coli* DH5α and plated on LB low salt supplemented with zeocin (25 μg/mL). Positive transformants were selected and the corresponding plasmids were extracted using GeneJET Plasmid Miniprep Kit (ThermoFisher Scientific, Waltham, MA, USA). All constructs were checked by sequencing (Macrogen Europe, Amsterdam, The Netherlands). Plasmids were linearized using PmeI for 3 h at 37 °C, purified, and transformed into *Pichia pastoris* X-33 by electroporation according to the EasySelect^TM^ *Pichia* expression kit guidelines (Life Technologies, Carlsbad, CA, USA). Positive transformants were selected on yeast extract peptone dextrose (YPD) plates supplemented with zeocin (100 μg/mL). The recombinant production and subsequent His_6_-tag purification of the fucosidase variants were performed as described previously [7].

### 2.5. Transglycosylation with Xyloglucan and Lactose

Transglycosylation activity was determined as described previously [7] using the xyloglucan-rich fraction extracted from citrus peel as a donor substrate with a concentration of bound Fuc of 2 mM. Lactose was used as an acceptor substrate at 100 mM. The reaction took place in 100 mM acetate buffer (pH 4.6) at 40 °C using 2.5 μM enzyme and was monitored for up to 24 h. At relevant time points, reaction samples were mixed 1:1 with preheated MilliQ water (95 °C) and kept at 95 °C for 10 min to stop the reaction. Initial reaction rates were calculated from the linear range of the product formation curves, which was up to 30–60 min for most variants, but in a few cases 10 min for high-activity variants and up to 24 h for low-activity ones.

The samples were analyzed by high-performance anion exchange chromatography with pulsed amperometric detection (HPAEC-PAD) on a Dionex ICS3000 system (Dionex, ThermoFisher Scientific, Waltham, MA, USA) using a CarboPac^TM^ PA1 analytical column (4 mm × 250 mm) equipped with a CarboPac^TM^ PA1 guard column (4 mm × 50 mm; Dionex, ThermoFisher Scientific, Waltham, MA, USA). Elution took place at a flow rate of 1 mL/min using an eluent system of MilliQ water (A), 500 mM NaOH (B), and 500 mM NaOAc with 0.02% (*w*/*v*) NaN_3_ (C). Fuc, 2′FL, and 3FL were used as external standards on a lactose background resembling that of the samples. For sample analysis, isocratic elution at 95:5:0 (% A:B:C) took place for 35 min. Strongly retained anions were washed from the column by elution with 15:5:80 (% A:B:C) for 5 min followed by re-equilibration of the column at 95:5:0 (% A:B:C) for 10 min.

Further identification of the fucosyllactose transfucosylation products was performed by liquid chromatography electrospray ionization mass spectrometry (LC–ESI–MS) on an Amazon SL iontrap (Bruker Daltonics, Bremen, Germany) coupled to an UltiMate 3000 UHPLC (Dionex, Sunnyvale, CA, USA) equipped with a porous graphitized carbon column (Hypercarb PGC, 150 mm × 2.1 mm; 3 μm, Thermo Fisher Scientific, Waltham, MA, USA) essentially as described previously [7], but with a smart parameter setting of 487 *m*/*z*. The formate adduct of 2′FL and its regioisomer were quantified against an external 2′FL standard curve by HPAEC-PAD.

### 2.6. Statistics 

One-way ANOVA for determination of statistical significance was performed with JMP Pro 14.1.0 (SAS Institute Inc., Cary, NC, USA). Statistical significance was established at *p* < 0.05.

## 3. Results and Discussion

### 3.1. Rational Design Based on the Previous Results

Directed evolution by random mutagenesis can be a powerful tool for identifying mutations, which improve transglycosylation significantly [13,24,33]. However, the fact that neither *Fg*FCO1 nor any GH29B members are active on the chromogenic substrate *p*NP-α-l-Fuc [7] complicates the screening process, since chromogenic substrates are most often used in the first mutant screening [13,22,23,24]. Instead, we aimed to transfer successful engineering results obtained on other GH29 α-l-fucosidases—including the GH29A *Tm*αFuc from *Thermotoga maritima*, which is active on *p*NP-α-l-Fuc—to the xyloglucan-active *Fg*FCO1 by rational design.

**Figure 1:**
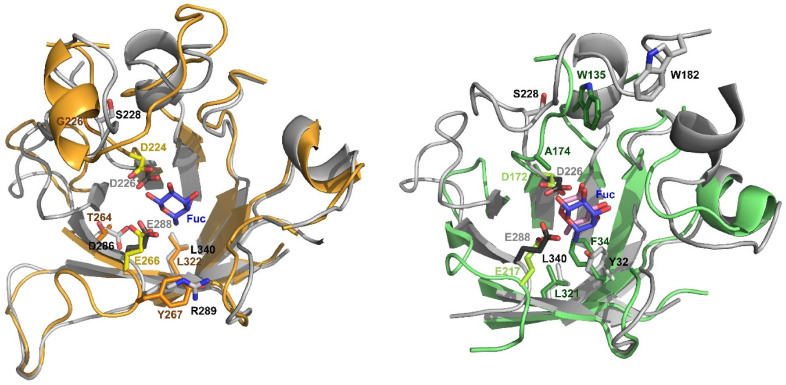
Active site structural alignments of *Fg*FCO1 (grey; PDB 4NI3) with *Tm*αFuc (orange; model on PDBs 1HL9 and 2ZWY) and *Bi*AfcB (green; PDB 3UES) indicating catalytic residues (dark grey with grey labels in *Fg*FCO1, yellow in *Tm*αFuc, and lime green in *Bi*AfcB). Residues that upon mutation had the most beneficial effect on transglycosylation in *Tm*αFuc [13] and *Bi*AfcB [6,34] are indicated in orange (*Tm*αFuc; brown labels) or green (*Bi*AfcB; green labels) alongside the corresponding residues if *Fg*FCO1 (grey with black labels). Corresponding *Fg*FCO1 residues were determined from multiple sequence alignment (Figure S1) guided by structural alignment around the catalytic acid/base (E288 in *Fg*FCO1), where sequence alignment is generally poor in GH29 [36]. Fuc ligands from *Fg*FCO1 (blue; PDB 4PSR) and *Bi*AfcB (pink; PDB 3UET) are indicated. Residues more than 14 Å away from the ligand are hidden for simplicity (with a few manual modifications). In the alignment of *Fg*FCO1 and *Tm*αFuc (left) amino acids 45-76 (*Tm*αFuc) and 46-52 (*Fg*FCO1) are also hidden as they block the view of the active site. The opening of the active site is facing the reader.

Indeed, the first example of engineering a GH29 α-l-fucosidase for improved transglycosylation was the work on the GH29A *Tm*αFuc, where directed evolution was combined with rational combinations of the identified mutation sites [13]. Using error prone PCR, around 5000 clones were screened for decreased hydrolytic activity. From these, approximately 100 colonies were selected and tested for transglycosylation activity. The best mutants were identified and rationally combined as 11 different enzyme variants, which all exhibited a higher ratio between transglycosylation and hydrolysis rates (*r*_T_/*r*_H_) than the WT. Three important second shell residues were identified: T264 and Y267 surrounding the catalytic acid/base E266, and L322, which is highly conserved throughout GH29 [13] (Figure 1 and Figure S1).

Furthermore, G226, two positions downstream of the catalytic nucleophile D224, appeared as a mutation accidentally introduced in the best two mutants, but no effect of this mutation was found [13]. In the TIM barrel structure, D224 and G226 are on the loop right after β4, T264 is on β6, E266 is at the end of β6, Y267 is on the loop right after β6, while L322 is on β8 (Figure 1). The best mutants were the quadruple mutants G226S-Y237H-T264A-L322P (where Y237 is far from the active site and was shown to have no effect) and G226S-T264A-Y267F-L322P [13].

The second example of GH29 engineering attempted to transfer T264A, Y267F, and L322P from *Tm*αFuc to GH29B *Bi*AfcB from *Bifidobacterium longum* subsp. *infantis* [6]. Structural alignment of the two enzymes, which belong to different subfamilies, revealed that only the highly conserved L322 (L321 in *Bi*AfcB) aligned perfectly. Consequently, only this mutation was tested in *Bi*AfcB. Instead, other conserved residues around subsite -1 were also targeted, namely first shell residues H36 and Y131 and second shell residues F34, E46, W170, and F291. Testing a mix of conservative mutations and more drastic ones, only F34 mutation resulted in significant increases in transglycosylation activity. Of the six variants tested (A, H, I, S, W, and Y), F34I was the best when combined with L321P [6]. F34 is on β1 in the TIM barrel structure (Figure 1).

No individual effect on transfucosylation was observed for the accidental mutation G226S in *Tm*αFuc, despite it being present in the two best mutants [13]. The corresponding residue two positions downstream of the catalytic nucleophile is A174 in *Bi*AfcB. The structural alignment between *Tm*αFuc and *Bi*AfcB is mediocre on this part of the loop, and as expected from the *Tm*αFuc study the single mutant A174S showed poor transglycosylation performance, although better than the WT [34]. However, the double mutant W135E-A174F stood out as highly potent in transglycosylation among many other variants, and markedly better than the single mutant W135E [34]. W135 is highly conserved in the multiple sequence alignment (Figure S1). A few additional conserved residues can be identified across GH29, but these are all first shell residues (Table 1). In general, second shell residues are more interesting targets in the conservative residues strategy [6,35], so no further residues were included as mutation targets in the rational design of *Fg*FCO1. Similarly, the loop structure targeted in the engineering of GH29B *Bb*AfcB [8] is not found in GH29A *Fg*FCO1 and the strategy was therefore not pursued in the current work. With previous successful mutations mapped (Table 1), rational design by mutation transfer to *Fg*FCO1 could commence. The aim of the study was to test the simplest method, namely mutating to the same residue as in previous studies despite differences in the WT sequence. Indeed, *Fg*FCO1 (GenBank AFR68935.1) has 25% sequence identity with *Tm*αFuc (GenBank AAD35394.1) and 31% sequence identity with *Bi*AfcB (GenBank ACJ53394.1), which in turn have 26% sequence identity with each other.

The main criterion for the selection of mutations to be transferred was structural alignment of the residues in question in the WT enzymes. *Tm*αFuc, *Bi*AfcB, and *Fg*FCO1 all have solved crystal structures [25,28,38], thus ensuring high credibility of structural alignment studies. Aligning *Fg*FCO1 to *Tm*αFuc and *Bi*AfcB, respectively, shows good alignment for L321 in *Bi*AfcB and L322 in *Tm*αFuc to L340 in *Fg*FCO1, which is placed on β8 (Figure 1). Similarly, T264 and Y267 in *Tm*αFuc align well with D286 and R289 in *Fg*FCO1, respectively (Figure 1). For G226 in *Tm*αFuc the alignment with S228 in *Fg*FCO1 was mediocre (3.4 Å between *C*_α_s). Interestingly, *Fg*FCO1 already has a serine in this position, so the G226S mutation became irrelevant. Alignment of S228 in *Fg*FCO1 to the corresponding residue A174 in *Bi*AfcB was not convincing with 4.7 Å between the *C*_α_s (Figure 1).

To test if a mutation transfer is still viable, the mutation S228F was included in this study. As may be expected, alignment of the loops following the TIM barrel β-strands is indeed not as good between GH29 subfamilies (*Fg*FCO1 vs. *Bi*AfcB) as the alignment within the same subfamily (*Fg*FCO1 vs. *Tm*αFuc). Indeed, while the tryptophan residue corresponding to W135 in *Bi*AfcB is highly conserved in the multiple sequence alignment (Figure S1), it is not aligned in the structural alignment of *Fg*FCO1 and *Bi*AfcB with a difference between the *C*_α_s of 14.5 Å (Figure 1). For *Tm*αFuc aligned with *Bi*AfcB, the distance between *C*_α_ of W178 and W135, respectively, was 6.8 Å, indicating that high sequence conservation did not appear to result in structural conservation in this position. Consequently, this position was not selected for mutation in *Fg*FCO1, being far from the active site (Figure 1). Another conserved residue is F34 on β1 in *Bi*AfcB, which corresponds to Y32 in *Fg*FCO1; phenylalanine is common in this position (Figure S1). Being on a TIM barrel β-sheet, structural conservation is high in this position (Figure 1).

In summary, the following mutations were chosen for *Fg*FCO1: D286A-R289F-L340P and D286A-L340P corresponding to the two best mutants previously obtained for *Tm*αFuc, Y32I-L340P, and S228F corresponding to the two superior mutants previously obtained for *Bi*AfcB, excluding the W182 residue due to poor structural alignment. In addition, all the single mutants and relevant double mutants were produced and tested for transglycosylation and hydrolytic activity.

### 3.2. Effect of Direct Mutation Transfer on Transglycosylation

The transfucosylation reaction using fucosylated xyloglucan extracted from citrus peel as the donor substrate and lactose as the acceptor substrate was monitored for 24 h. The resulting initial rates of Fuc release and 2′FL synthesis were determined alongside the molar yield of 2′FL on the fucosyl donor (Table 2). For efficient glycosidase-catalyzed transglycosylation to take place, the ratio between transglycosylation and hydrolysis should be maximized. This is often expressed as the ratio between the initial rates of formation, *r*_T_/*r*_H_ (Table 2). Unlike previously reported [7], formation of smaller amounts of another fucosyllactose regioisomer was observed. Its molecular mass corresponded to fucosyllactose (Figure S2), and based on its retention time on HPAEC-PAD, which was close to that of 2′FL and far from that of 3FL, we speculated that this is a regioisomer fucosylated on either *O*-3, *O*-4, or *O*-6 of the non-reducing end Gal moiety and denote it X’FL. The initial rate of formation and the molar yield of X’FL were determined (Table 2). Low regioselectivity is a general issue in GH29A [26].

Certain attempts at direct mutation transfer from previous studies resulted in complete loss of function. S228 is close to the catalytic nucleophile D226 in an area where both sequence and structural alignments are poor (Figure 1 and Figure S1). The mutation S228F resulted in complete loss of activity (Table 2), indicating that rational design by mutation transfer is not straightforward in a poorly aligned area, or at least not so simple that the best mutant from another subfamily can be directly transferred. The best directed evolution mutants of *Tm*αFuc did indeed include the mutation G226S, indicating that a Ser in this position is not at all bad for transfucosylation activity. In *Bi*AfcB, A174S also increased transfucosylation activity significantly compared to the WT, although not as much as the best variants, which included A174F [34].

The mutation R289F resulted in an almost complete loss of function with only negligible amounts of Fuc being detected in the reaction and no transglycosylation products (Table 2; Figure S3). In the combination D286A-R289F, all products were below the detection level (Table 2; Figure S3). From the crystal structures of *Fg*FCO1 it was evident that R289 and several other amino acids on the 287-WERG-290 loop changed conformation between the open and closed forms of the enzyme. In the closed form, R289 interacts with W311 as the 287-WERG-290 loop moves towards the active center, whereas R289 interacts with F294 away from the active center in the open form [25]. Thus, the mutation R289F might have disturbed this conformational change, which may play a role in the catalytic function of *Fg*FCO1. In addition, whereas the mutation Y267F in *Tm*αFuc is of the conservative kind (Table 1), a change from Arg to Phe is more dramatic.

In contrast to R289, D286 does not change conformation between the open and closed forms of *Fg*FCO1 [25]. Mutation of this residue two positions upstream from the catalytic acid/base to D286A (similar to T264A in the best *Tm*αFuc mutants) resulted in significantly increased initial rates of formation for all reaction products (Table 2). However, the rates increased more for Fuc and X’FL than for 2′FL. As a result, the *r*_T_/*r*_H_ and the yield of 2′FL were lower than for the WT (Table 2). Since 2′FL is also a substrate of *Fg*FCO1 [7], increased hydrolytic activity may cause inadvertent product hydrolysis. Indeed, the increased hydrolytic activity resulted in a decrease in 2′FL yield after 3 h of reaction, while the amount of X’FL kept increasing throughout the 24 h (Figure S3).

Previously, the mutation of the highly conserved Leu residue corresponding to L340 in *Fg*FCO1 to Pro has improved transfucosylation in both GH29A and GH29B [6,13]. Being conserved in sequence and structure across both GH29 subfamilies, this should be an ideal mutation to increase transglycosylation efficiency for all members of GH29. However, for *Fg*FCO1 the introduction of L340P had a severely detrimental effect on the recombinant enzyme expression in *P. pastoris*. Consequently, the activity of *Fg*FCO1 variants D286A-R289F-L340P, D286A-L340P, and L340P could not be determined (Table 2). Only *Fg*FCO1 Y32I-L340P was produced in adequate amounts, yet still very low.

Mutation of another fairly conserved residue, Y32I improved the regioselectivity of *Fg*FCO1 (Table 2). However, both rates, *r*_T_/*r*_H_ and yields decreased significantly compared the WT (Table 2; Figure S3). Combining Y32I with L340P (similar to F34I-L321P of *Bi*AfcB) did not improve rates or yields (Table 2), but was instead detrimental to expression levels. In a combination of the two point mutations with most positive effects, i.e., D286A and Y32I, all reaction rates were slightly, though not significantly, increased compared to Y32I (Table 2). However, as also observed for D286A the rate of hydrolysis increased most, resulting in a lower *r*_T_/*r*_H_ and slightly lower yield of 2′FL, while at the same time compromising regioselectivity (Table 2). For the reactions with *Bi*AfcB F34I-L321P, the enzyme concentrations are not disclosed or yet optimized [6], but it is often seen that higher enzyme dosages are required to obtain superior transglycosylation yields with enzyme variants where conserved residues have been mutated [37]. In the current work, equimolar dosage was used for an equal comparison. Possibly, higher product yields could have been obtained with a higher dosage of *Fg*FCO1 Y32I or Y32I-L340P.

In conclusion, direct mutation transfer is not straightforward when structural or sequence alignment is poor, at least not when employing the simplest possible method of introducing the same amino acid as reported for other enzymes. Interestingly, the most potent mutation identified in this work originates from a study employing directed evolution, and not from targeting conserved residues. This emphasizes the importance of a robust analytical system for high-throughput screening of variants for transglycosylation reactions. However, the mutations that stood out in the directed evolution study on *Tm*αFuc [13] appear far from random: In fact, the most important ones were either very close to the catalytic residues (1–2 positions away) or among conserved residues (L322P). This knowledge, combined with the data for *Bi*AfcB [6,34] and those obtained here for *Fg*FCO1 (Table 2) could facilitate the design of smaller mutant libraries for protein engineering targeting residues close to the catalytic residues and conserved ones.

While none of the *Fg*FCO1 variants outcompeted the WT in terms of *r*_T_/*r*_H_ or transfucosylation yield, the increased reaction rate obtained with D286A was an important result, which could lead to more efficient biocatalysis, and notably help pinpoint the significance of partially distal residues in the catalytic function of the enzyme and perhaps even illuminate how the *r*_T_/*r*_H_ balance is controlled. Keeping in mind that simply copying the amino acid substitution reported for other enzymes may not be a viable strategy, mutation of D286—the amino acid residue two positions upstream from the proposed catalytic acid/base E288—was studied further through site-saturation mutagenesis (SSM).

### 3.3. Site-Saturation Mutagenesis (SSM) of D286

D286 is a second shell residue approximately 7 Å away from the Fuc ligand and as its mutation is expected to cause conformational changes in the active site rather than to interfere directly with substrate binding. However, homology modeling of the D286 SSM variants did not reveal any major changes in the active site upon substitution of D286 (Figure S4). In the homology models of several variants including D286M, D286R, and D286I, a movement was observed for the side chain of W224, which interacts directly with D286. W224 is two positions upstream of the catalytic nucleophile and 3–4 Å away from the Fuc ligand; thus, some effect on activity could be expected. In some cases, the catalytic acid/base E288 and especially F394 are found in different conformations or positions when comparing the models of the variants to the WT *Fg*FCO1 model (Figure S4). However, these residues are known to be highly flexible and found in different conformations in the open and closed forms of *Fg*FCO1, respectively [25]. For the D286M variant, a conformational change of F227 adjacent to the catalytic nucleophile is observed (Figure S4). This residue was however approximately 9 Å away from D286 and its conformational change was therefore not an obvious result of the D286M mutation. 

Initial rates of formation (*r*) of Fuc, 2′FL, and X’FL were determined for all D286 SSM variants alongside molar yields of 2′FL and X’FL over the 24-h course of reaction (Table 3). Introduction of the rigid Pro or the aromatic Phe and Tyr at this position resulted in complete loss of activity (Table 3). Similarly, the lowest detected product yield was obtained with D286W (Table 3), indicating that introduction of an aromatic side chain in this position is—not surprisingly—detrimental to the overall activity. However, substitution of D286 with His had a positive effect on the rates of formation compared to the WT. Although the maximum product yields were not significantly different between WT and D286H (Table 3), the higher rates of formation meant that at shorter reaction times, the yields were higher for D286H than for WT *Fg*FCO1, e.g., after 1 h of reaction the 2′FL yield was 7.8% ± 0.8% for D286H and 3.1% ± 0.3% for WT (Figure S5). This could be linked to the positive charge of His, since D286R exhibited initial rates of formation and yields similar to those of D286H, except for formation of X’FL, which was markedly higher for D286H than for D286R (Table 3; Figure S5). However, the effect of a positively charged amino acid in this position is not universal, seeing as D286K had very low activity (Table 3). 

Substituting the negatively charged D286 with the slightly larger Glu did not change the initial rate of formation of 2′FL significantly. However, both *r*_H_ and *r*_X’FL_ increased, resulting in lower yields of 2′FL due to product hydrolysis and poorer regioselectivity than observed for WT *Fg*FCO1 (Table 3; Figure S5). Substituting the carboxylic acid with amides in D286N and D286Q had a detrimental effect on all rates and yields; again, the effect was worse with the larger of the two side chains (Table 3; Figure S5).

As described above, the substitution of D286 with the small, hydrophobic Ala resulted in an enzyme variant with higher reaction rates, although more so for the hydrolytic activity than for transglycosylation (Table 2 and Table 3). Substituting with the even smaller Gly had a detrimental effect on all reaction rates and yields compared to the WT (Table 3).

Mixed effects were observed when substituting D286 with the aliphatic amino acids. For both D286I and D286V, initial rates of formation of Fuc and 2′FL were significantly improved compared to the WT with *r*_2′FL_ being among the highest observed (Table 3). For D286V, *r*_X’FL_ was also significantly increased, which was not the case for D286I (Table 3). Among the three variants, the highest 2′FL yield was obtained with D286I (9.5% ± 2%), which was however not higher—nor significantly lower—than that of the WT (11% ± 5%). However, the maximum yield for D286I was obtained after 5 h of reaction, where the WT yield was at 9.7% ± 1%. At even shorter reaction times, higher yields of 2′FL were obtained with D286I than with WT *Fg*FCO1, e.g., 7.2% ± 0.8% for D286I after 1 h of reaction (Figure S5). On the other hand, substitution with Leu, which is the largest of the three aliphatic amino acids, was detrimental to activity: although not statistically significant, all initial rates of formation were lower than those of the WT, and the product yield was significantly lower. However, as also observed for D286I and D286V, the regioselectivity was improved compared to the WT as no formation of X’FL could be detected with D286L (Table 3).

Thr and Val are similar in size and structure, and despite different polarity, these amino acid substitutions at D286 had similar effects on *r*_H_ and *r*_X’FL_ resulting in significantly higher initial rates of formation for Fuc and X’FL, which were similar for D286T and D286V (Table 3). Between the two, the non-polar Val had a significantly more positive effect on formation of 2′FL, and as a result, the *r*_T_/*r*_H_ and the regioselectivity were much better for D286V than for D286T (Table 3). In fact, the yield of X’FL was higher than the 2′FL yield for D286T (Table 3). The same was observed when substituting D286 with the small, polar Ser. D286S exhibited slightly (but not significantly) increased initial rates of formation for Fuc and X’FL, but this mutation was severely detrimental to 2′FL formation, leading to a low 2′FL yield and poor regioselectivity (Table 3).

Finally, introduction of the sulfur-containing Cys led to increased initial reaction rates, but only significantly so for formation of Fuc and X’FL. As a result, *r*_T_/*r*_H_ was low and the 2′FL yield did not exceed 3.2% ± 0.4% (Table 3). In contrast, substitution with Met increased initial rates of formation 2.4-fold compared to the WT for both Fuc and 2′FL, whereas the rate of X’FL formation was largely unchanged. This resulted in the highest 2′FL yield obtained (16% ± 6%) and a better regioselectivity than observed for the WT. Thus, D286M was a significant improvement to *Fg*FCO1 (Table 3). However, none of the substitutions of D286 caused dramatic improvements in the transfucosylation yield.

SSM studies are scarce for protein engineering studies aiming to improve transglycosylation efficiency. However, a recent study on the GH20 β-*N*-acetylhexosaminidase BbhI performed SSM on two positions. At both positions, substitutions with amino acids, which were chemically quite different from the WT amino acids, were observed as the best mutants, namely BbhI D714T and W773R [24]. For D714, more than half of the variants were completely inactive, whereas all substitutions of W773 resulted in active mutants, with most of them reaching higher product yields than WT BbhI [24]. In this light, it is less surprising that the fairly radical change from Asp to Met in *Fg*FCO1 D286M is the best mutant, and that several other mutants also show increased reaction rates as compared to the WT. Together, these studies emphasize the benefits of not only introducing conservative mutations in protein engineering.

### 3.4. Improved Transglycosylation by Substrate Depolymerization 

It was hypothesized that depolymerization of the xyloglucan fucosyl donor substrate would improve transglycosylation yields due to improved enzyme access. The size distribution of the extracted citrus peel xyloglucan showed a distribution across the range from 5 to 110 kDa (Figure S6). Upon treatment with the GH5 *Paenibacillus* sp. xyloglucan-specific endo-β-1,4-glucanase, significant depolymerization took place, resulting in a size distribution with a major peak below 5 kDa, a medium peak around 12 kDa, and a smaller peak around 110 kDa (Figure S6).

All D286 variants with an initial rate of formation of 2′FL significantly higher than that of WT *Fg*FCO1 were selected for determining transglycosylation activity using the depolymerized xyloglucan donor substrate, namely D286H, D286I, D286M, D286R, and D286V. In addition, D286A, D286C, and D286E all had slightly, but not significantly, higher *r*_2′FL_ than the WT; of these, the two with the highest *r*_T_/*r*_H_ were included in the study: D286A and D286E.

Compared to the reaction on the untreated substrate, yields of 2′FL increased 2.6-fold for D286E, 2.5-fold for D286R and D286H, 2.2-fold for D286V, 1.8-fold for D286I, and 1.6-fold for WT *Fg*FCO1 when used with the XEG-treated donor substrate. The slight increases for D286M (1.2-fold) and D286A (1.4-fold) were not statistically significant. Nevertheless, the results emphasize that the XEG-catalyzed depolymerization of the fucosylated xyloglucan substrate consistently improved 2′FL yields. The highest 2′FL yields were obtained with D286H (23% ± 1%) and D286R (22% ± 0.6%), closely followed D286M (20% ± 1%). Slightly lower yields were obtained with WT (18% ± 0.7%), D286I (17% ± 1%), and D286V (16% ± 1%). With D286A and D286E, which were also the least efficient of these selected variants in the SSM study (Table 3) markedly lower yields were obtained (Table 4).

All initial rates of formation were significantly higher on the XEG-treated substrate, indicating that the depolymerization improved catalysis, most likely through increased enzyme access. The *r*_2′FL_ increased 3–5 times compared to those obtained on the untreated substrate, with the highest increases observed for WT, D286H, and D286M. The highest *r*_2′FL_ values were obtained for D286H and D286R, which were also the variants to reach the highest 2′FL yields, despite the fact that D286R also exhibited the highest *r*_H_ (Table 4). Indeed, significant product hydrolysis was observed for D286R and the maximum yield was reached within 30 min of reaction (Figure S7). In fact, product hydrolysis was markedly higher for all *Fg*FCO1 variants when using the XEG-treated substrate (Figure S7): Transient maximum yields of 2′FL were reached within 30 min of reaction for D286H, D286R, and D286V, after 1 h of reaction for D286E, D286I, and D286M, after 2 h for D286A, and after 3 h for WT *Fg*FCO1.

Finally, donor substrate depolymerization also had a positive effect on regioselectivity for all tested variants except D286A (Table 3 and Table 4), possibly due to the high formation rates for 2′FL and the short reaction times required to reach the transient maxima. As the rates of formation for X’FL were much lower, and generally increased less than *r*_2′FL_ on the XEG-treated substrate compared to the untreated donor substrate, the ratio between the 2′FL and X’FL concentrations increased at the time of maximum 2′FL yield. As also observed on the untreated substrate, the highest regioselectivity was obtained with D286M and D286I (Table 3 and Table 4). For D286M, the X’FL yield was merely 0.4% after 1 h of reaction, where the maximum 20% yield of 2′FL was obtained (Table 4; Figure S7).

Despite the improved substrate accessibility achieved through depolymerization, the total product yield (including free Fuc) never exceeded 70% of the theoretical maximum (Figure S7), which is based on the Fuc content measured from acid hydrolysis of the extracted xyloglucan-rich substrate [7]. This indicates that approximately 30% of the bound Fuc remains inaccessible to *Fg*FCO1, which naturally affects the maximum achievable transglycosylation yield. This may be explained by the Fuc-substituted side chains curling around the glucan backbone [39] in a less accessible position, or because some of the Fuc is bound in internal or substituted positions in rhamnogalacturonan-II, which are inaccessible to members of GH29 [40], rather than as terminal α-l-Fuc in xyloglucan.

In conclusion, the simple XEG-treatment of the fucosylated xyloglucan donor substrate proved to be an efficient method for increasing fucosyllactose yields in the current reaction setup. It has previously been observed that large donor substrates cause mass transfer limitations in transglycosylation reactions [41]. On the XEG-treated substrate, D286H and D286R gave significantly higher yields than WT *Fg*FCO1. While a significant increase in the 2′FL yield compared to the WT was observed for D286M on the untreated substrate, the increase also observed for D286M on the XEG-treated substrate was not statistically significant (Table 4).

However, considering product yields in combination with regioselectivity towards formation of 2′FL, D286M outperformed the other *Fg*FCO1 variants and the WT on both donor substrates (Table 3; Table 4). Importantly, the increase in reaction yields obtained by substrate depolymerization was more dramatic than the one obtained by protein engineering.

### 3.5. Confirmation of E288 as the Catalytic Acid/Base in FgFCO1

The crystal structure of *Fg*FCO1 clearly indicated D226 and E288 as the catalytic nucleophile and the catalytic acid/base, respectively [25]. While the catalytic nucleophile (Asp) is highly conserved in sequence alignments of GH29 members, the catalytic acid/base is not as easily assigned from the sequence [36] (Figure S1). In order to better understand the effect of the SSM studies on the residue D286 in the current work, it was important to confirm the catalytic function of the nearby residue E288 by constructing inactive mutants. As a control, similar mutants of the nearby E234 were included. In order to confirm the catalytic function for the WT and for selected D286 variants, the following mutations were made: E234A, E288A, E234A-D286R, D286R-E288A, E234A-D286M, and D286M-E288A. The resulting *Fg*FCO1 variants were tested for transglycosylation activity using untreated xyloglucan as the donor substrate. The initial rates of formation were approximately doubled for E234A and E234A-D286M compared to their counterparts WT and D286M: For formation of 2′FL, the initial rates were 1.0 ± 0.04 and 2.5 ± 0.5 μmol min^−1^ (μmol E)^−1^ for E234A and E234A-D286M, respectively. For Fuc formation, the initial rates were 0.44 ± 0.2 and 1.35 ± 0.7 μmol min^−1^ (μmol E)^−1^ for E234A and E234A-D286M, respectively. Using E234A-D286R, the rates of formation were similar to those of D286R (0.76 ± 0.2 μmol min^−1^ (μmol E)^−1^ for Fuc and 0.93 ± 0.2 μmol min^−1^ (μmol E)^−1^ for 2′FL). In contrast, no detectable activity was observed for E288A, D286R-E288A, and D286M-E288A, confirming that E288 is indeed the catalytic acid/base residue for *Fg*FCO1 WT and for the selected D286 mutants.

## 4. Conclusions

Rational design by direct mutation transfer between members of the same GH family often works well for conserved residues—even across GH subfamilies—as observed for F426Y of CelB from *Pyrococcus furiosus* and F441Y of LacS from *Sulfolobus solfataricus* in GH1 [27] and for L322P of *Tm*αFuc and L321P of *Bi*AfcB in GH29 [6]. Transfer of F34I in *Bi*AfcB to Y32I in *Fg*FCO1 is a new example of mutation transfer for a fairly conserved residue across GH29 subfamilies. Although not as efficient in transglycosylation as the WT at equimolar dosage, this mutation did have a positive effect on *Fg*FCO1 regioselectivity. Transferability between the subfamilies in GH29 is tightly linked to conservation, which is in turn often—but not always—reflected in high structural alignment; both parameters could be used when selecting new mutation targets.

As an alternative to conserved residues, amino acids, which are close to the catalytic residues in the sequence, could be targeted. The present results on D286 substitution emphasize that small changes in the active site should be among future targets for engineering. Indeed, several other amino acids in this position resulted in a more efficient biocatalyst, both in terms of reaction rates and transglycosylation yields. D286 is positioned on a β-sheet in the central TIM barrel and is structurally conserved. For other residues in areas where structural and/or sequence alignment was poor, direct mutation transfer was not possible in *Fg*FCO1 and resulted in loss of activity. As emphasized recently, protein engineering of GH29A α-l-fucosidases for improved regioselectivity is required to obtain better results in transglycosylation for synthesis of α-1,2-fucosylated structures [26]. While not completely regioselective, *Fg*FCO1 D286M represents a step in the right direction, both in terms of product yields and especially regioselectivity.

Finally, enzymatic depolymerization of the large donor substrate resulted in dramatically improved reaction rates and in turn higher product yields. However, the xyloglucan depolymerization also demanded a closer control of the reaction time since transient product maxima occurred as a result of the increased reaction rates. Nevertheless, such substrate depolymerization should be considered as a go-to method in all enzymatic transglycosylation processes involving large, polymeric substrates.

## Figures and Tables

**Table 1:** Overview of catalytic residues, highly conserved residues, and other relevant amino acid residues and their respective numbering (#) in the three GH29 α-l-fucosidases discussed in this work: *Tm*αFuc, *Bi*AfcB, and *Fg*FCO1 (the latter using numbering from the crystal structure [25]). The assignment of similar residues is generally deduced from sequence alignment, except for the area around the catalytic acid/base where sequence alignment is generally poor [36]; assignments here were guided by crystal structures (Figure S1). Mutations reported for each residue are included; an asterisk (*) designates mutations, which improved transglycosylation. Conserved residues were determined from multiple sequence alignment (Figure S1); all G and P residues are excluded as it is generally not advised to mutate these due to their involvement in protein folding [37].

Relevance	*Fg*FCO1 #	*Tm*αFuc #	*Bi*AfcB #	Mutations Reported *^a^*
**Catalytic residues**				
Nucleophile	D226	D224	D172	-
Acid base	E288	E266	E217	-
**Conserved residues *^b^***				
Second shell (close to first shell in *Fg*FCO1)	Y32	F32	F34	F34I best in *Bi*AfcB double mutant [6] * Y32I in *Fg*FCO1 (this work)
First shell	H34	H34	H36	H36N/I in *Bi*AfcB [6]
First shell in *Tm*αFuc and *Fg*FCO1; second shell in *Bi*AfcB	E46	E66	E46	E46Q/I in *Bi*AfcB [6]
First shell	W47	W67	W47	-
First shell	H126	H128	H85	-
First shell	H127	H129	H86	-
First shell in *Bi*AfcB, second shell in *Tm*αFuc	Y176 *^c^*	Y171	Y131	Y131IF in *Bi*AfcB [6]
Second shell in *Bi*AfcB and *Tm*αFuc; further away (13 Å) in *Fg*FCO1 (Figure 1)	W182	W178	W135	W135E best in *Bi*AfcB double mutant (SSM) [34] *
Second shell	W224	W222	W170	W170F/A in *Bi*AfcB [6]
First shell in *Tm*αFuc and *Fg*FCO1; second shell in *Bi*AfcB	W311	F290	F291	F291S in *Bi*AfcB [6]
Second shell; identified in *Tm*αFuc by directed evolution	L340	L322	L321	L322P in *Tm*αFuc [13] * L321P in *Bi*AfcB [6] * L340P in *Fg*FCO1 (this work)
**Other relevant residues**				
Identified in *Tm*αFuc by directed evolution	S228	G226	A174	G226S in *Tm*αFuc [13] A174F best in *Bi*AfcB double mutant (SSM) [34] * S228F in *Fg*FCO1 (this work)
Identified in *Tm*αFuc by directed evolution	D286	T264	G215	T264A in *Tm*αFuc [13] * SSM in *Fg*FCO1 (this work)
Identified in *Tm*αFuc by directed evolution	R289	Y267	A218	Y267F in *Tm*αFuc [13] * R289F in *Fg*FCO1 (this work)

*^a^* More mutations have been introduced in *Bi*AfcB, several of which were introduced for improving thermal stability [34]. These are beyond the scope of the work. Consequently, only those reasonably close to the active site (<9 Å from the ligand in PDB 3UET) are included here. *^b^* Only conserved residues surrounding the active site are given; as expected, they are all found around subsite-1. D152 in *Tm*αFuc = D150 in *Fg*FCO1 = D112 in *Bi*AfcB is also highly conserved in GH29, but is placed very far from the active site. SSM = site-saturation mutagenesis. *^c^* Both sequence and structural alignment are poor in this position for *Fg*FCO1.

**Table 2:** Transfucosylation with rationally designed variants of *Fg*FCO1. Initial rates of formation (*r*) of fucose (Fuc), 2′-fucosyllactose (2′FL), and its regioisomer X’FL in the transfucosylation catalyzed by *Fg*FCO1 variants using fucosylated citrus peel xyloglucan as the fucosyl donor and lactose as the acceptor over 24 h. The ratio between the transfucosylation rate (*r*_T_) and the hydrolysis rate (*r*_H_) is indicated: the first number is calculated for 2’FL alone, whereas the number in brackets includes the sum of FL isomers. Maximum molar yields on the donor substrate obtained during the 24 h of reaction are given for 2′FL and X’FLA few enzyme variants could not be expressed in detectable amounts (n.e.). For some enzyme variants, activity was below the HPAEC-PAD detection limit (b.d.) of 0.05 μM product, which equals a product yield of 0.0025%; these numbers were included in the statistical analysis. Superscript letters (*a*–*d*) indicate significant differences between enzyme variants for each rate of formation or for the yield of each product (*p* < 0.05). Regioselectivity ([2′FL]/[X’FL]) is calculated at the highest yield of 2′FL.

*Fg*FCO1 Variant	Initial Rate of Formation *r* (μmol min^−1^ (μmol E)^−1^)			*r*_T_/*r*_H_ (incl. X’FL)	Maximum Transfucosylation Yield [%]		Regioselectivity [2′FL]/[X’FL]
	Fuc	2′FL	X’FL		2′FL	X’FL	
WT	0.26 ± 0.09 *^b^*	0.46 ± 0.09 *^b^*	0.026 ± 0.01 *^b^*	1.8 (1.9)	11 ± 5 *^a^*	4.7 ± 2.3 *^a^*	2.4
D286A-R289F-L340P	n.e.						
D286A-L340P	n.e.						
D286A-R289F	b.d. *^d^*	b.d. *^c^*	b.d.*^c^*	-	b.d. *^c^*	b.d. *^b^*	-
L340P	n.e.						
D286A	0.66 ± 0.003 *^a^*	0.60 ± 0.01 *^a^*	0.078 ± 0.02 *^a^*	0.90 (1.0)	4.8 ± 0.7 ^bc^*	4.3 ± 0.5 ^a^*	2.5
R289F	0.004 ± 0.0002 *^d^*	b.d. *^c^*	b.d. *^c^*	-	b.d. *^c^*	b.d. *^b^*	-
Y32I-L340P	0.014 ± 0.0004 *^cd^*	0.011 ± 0.001 *^c^*	0.0017 ± 0.0001 *^c^*	0.79 (0.91)	5.2 ± 0.2 *^bc^*	0.3 ± 0.02 *^b^*	17
Y32I	0.033 ± 0.001 *^cd^*	0.034 ± 0.003 *^c^*	0.0023 ± 0.0005 *^c^*	1.0 (1.1)	6.5 ± 0.6 *^ab^*	0.4 ± 0.1 *^b^*	16
S228F	b.d. *^d^*	b.d. *^c^*	b.d.*^c^*	-	b.d. *^c^*	b.d. *^b^*	-
Y32I-D286A	0.12 ± 0.004 *^c^*	0.074 ± 0.01 *^c^*	0.017 ± 0.005 *^bc^*	0.64 (0.78)	4.6 ± 1 *^bc^*	4.1 ± 0.8 *^a^*	1.1

* D286A: The maximum yield of 2′FL obtained after 180 min, where the yield of X’FL was 1.9% (Figure S3). After 24 h, the 2′FL yield was 3.6% and X’FL 4.3%. All other variants in this table and the WT have maximum yields of all products after 24 h.

**Table 3:** Transfucosylation with D286 site-saturation mutagenesis (SSM) variants of *Fg*FCO1. Initial rates of formation (*r*) of fucose (Fuc), 2′-fucosyllactose (2′FL), and its regioisomer X’FL in the transfucosylation catalyzed by *Fg*FCO1 SSM variants of D286 using fucosylated citrus peel xyloglucan as the fucosyl donor and lactose as the acceptor over 24 h. The ratio between the transfucosylation rate (*r*_T_) and the hydrolysis rate (*r*_H_) is indicated: the first number is calculated for 2’FL alone, whereas the number in brackets includes the sum of FL isomers. Maximum molar yields on the donor substrate obtained during the 24 h of reaction are given for 2′FL and X’FL. For some enzyme variants, activity was below the HPAEC-PAD detection limit (b.d.) of the 0.05 μM product, which equals a product yield of 0.0025%; these numbers were included in the statistical analysis. Superscript letters (*a*–*g*) indicate significant differences between enzyme variants for each rate of formation or for yield of each product (*p* < 0.05). Regioselectivity ([2′FL]/[X’FL]) is calculated at the highest yield of 2′FL.

*Fg*FCO1 Variant	Initial Rate of Formation *r* (μmol min^−1^ (μmol E)^−1^)			*r*_T_/*r*_H_ (incl. X’FL)	Maximum Transfucosylation Yield (%)		Regioselectivity [2′FL]/[X’FL]
	Fuc	2′FL	X’FL		2′FL	X’FL	
WT	0.26 ± 0.09 *^ef^*	0.46 ± 0.09 *^cd^*	0.026 ± 0.01 *^g^*	1.8 (1.9)	11 ± 5 *^b^*	4.7 ± 2.3 *^ab^*	2.4
D286A	0.66 ± 0.003 *^bcd^*	0.60 ± 0.01 *^c^*	0.078 ± 0.02 *^cde^*	0.90 (1.0)	4.8 ± 0.7 *^cde^*	4.3 ± 0.5 *^abc^*	2.5
D286C	0.99 ± 0.02 *^b^*	0.58 ± 0.09 *^cd^*	0.090 ± 0.009 *^bcd^*	0.58 (0.67)	3.2 ± 0.4 *^cde^*	3.1 ± 0.6 *^bcd^*	2.3
D286E	0.77 ± 0.1 *^bc^*	0.57 ± 0.2 *^c^*	0.11 ± 0.02 *^bc^*	0.75 (0.89)	4.4 ± 2 *^cd^*	6.2 ± 0.3 *^a^*	2.8
D286F	b.d. *^f^*	b.d. *^f^*	b.d. *^g^*	-	b.d. *^e^*	b.d. *^e^*	-
D286G	0.20 ± 0.004 *^ef^*	0.083 ± 0.002 *^ef^*	0.011 ± 0.002 *^g^*	0.42 (0.48)	5.2 ± 0.1 *^cd^*	0.8 ± 0.1 *^de^*	6.3
D286H	0.60 ± 0.03 *^cd^*	1.3 ± 0.1 *^a^*	0.13 ± 0.05 *^b^*	2.1 (2.3)	9.3 ± 2 *^bc^*	5.0 ± 3 *^ab^*	2.2
D286I	0.69 ± 0.04 *^bcd^*	1.1 ± 0.2 *^ab^*	0.028 ± 0.02 *^fg^*	1.6 (1.7)	9.5 ± 2 *^bc^*	1.6 ± 2 *^de^*	9.5
D286K	0.018 ± 0.0005 *^f^*	0.020 ± 0.003 *^f^*	b.d. *^g^*	1.1 (1.1)	0.4 ± 0.1 *^de^*	b.d. *^e^*	-
D286L	0.11 ± 0.02 *^ef^*	0.34 ± 0.003 *^cde^*	b.d. *^g^*	3.1 (3.1)	3.5 ± 0.1 *^cde^*	b.d. *^e^*	-
D286M	0.64 ± 0.4 *^cd^*	1.1 ± 0.2 *^ab^*	0.032 ± 0.01 *^fg^*	1.7 (1.7)	16 ± 6 *^a^*	2.2 ± 0.2 *^cde^*	7.4
D286N	0.11 ± 0.003 *^ef^*	0.043 ± 0.003 *^f^*	b.d. *^g^*	0.38 (0.38)	4.6 ± 0.6 *^cde^*	b.d. *^e^*	-
D286P	b.d. *^f^*	b.d. *^f^*	b.d. *^g^*	-	b.d. *^e^*	b.d. *^e^*	-
D286Q	0.058 ± 0.007 *^ef^*	0.040 ± 0.01 *^f^*	b.d. *^g^*	0.7 (0.7)	1.1 ± 0.2 *^de^*	b.d. *^e^*	-
D286R	0.76 ± 0.3 *^bc^*	1.2 ± 0.3 *^a^*	0.062 ± 0.05 *^def^*	1.5 (1.6)	8.7 ± 2 *^bc^*	1.7 ± 1 *^de^*	6.4
D286S	0.38 ± 0.03 *^de^*	0.078 ± 0.005 *^ef^*	0.028 ± 0.002 *^efg^*	0.21 (0.28)	1.6 ± 0.2 *^de^*	4.4 ± 0.2 *^abc^*	1.5
D286T	1.4 ± 0.1 *^a^*	0.27 ± 0.04 *^def^*	0.19 ± 0.009 *^a^*	0.19 (0.32)	2.1 ± 1 *^de^*	6.3 ± 2 *^a^*	0.5
D286V	1.4 ± 0.2 *^a^*	0.99 ± 0.1 *^b^*	0.18 ± 0.03 *^a^*	0.70 (0.82)	7.1 ± 1 *^c^*	6.1 ± 0.9 *^a^*	5.9
D286W	0.052 ± 0.01 *^ef^*	0.005 ± 0.0004 *^f^*	b.d. *^g^*	0.10 (0.10)	0.3 ± 0.04 *^e^*	b.d. *^e^*	-
D286Y	b.d. *^f^*	b.d. *^f^*	b.d. *^g^*	-	b.d. *^e^*	b.d. *^e^*	-

**Table 4:** Transfucosylation with depolymerized xyloglucan donor substrate. Initial rates of formation (*r*) of fucose (Fuc), 2′-fucosyllactose (2′FL), and its regioisomer X’FL in the transfucosylation catalyzed by *Fg*FCO1 site-saturation mutagenesis variants of D286 using XEG-treated fucosylated citrus peel xyloglucan as fucosyl donor and lactose as an acceptor over 24 h. The ratio between the transfucosylation rate (*r*_T_) and the hydrolysis rate (*r*_H_) is indicated: the first number is calculated for 2’FL alone, whereas the number in brackets includes the sum of FL isomers. Maximum molar yields on the donor substrate obtained during the 24 h of reaction are given for 2′FL and X’FL.Superscript letters (*a*–*e*) indicate significant differences between enzyme variants for each rate of formation or for yield of each product (*p* < 0.05). Regioselectivity ([2′FL]/[X’FL]) is calculated at the highest yield of 2′FL.

*Fg*FCO1 Variant	Initial Rate of Formation *r* (μmol min^−1^ (μmol E)^−1^)			*r*_T_/*r*_H_ (incl. X’FL)	Maximum Transfucosylation Yield (%)		Regioselectivity [2′FL]/[X’FL]
	Fuc	2′FL	X’FL		2′FL	X’FL	
WT	0.88 ± 0.3 *^d^*	2.1 ± 0.2 *^c^*	0.063 ± 0.008 *^c^*	2.3 (2.4)	18 ± 0.7 *^cd^*	6.1 ± 1 *^cd^*	13
D286A	2.9 ± 0.7 *^ab^*	1.7 ± 0.4 *^c^*	0.44 ± 0.06 *^b^*	0.59 (0.74)	6.7 ± 1 *^f^*	10 ± 0.8 *^b^*	1.1
D286E	1.8 ± 0.3 *^cd^*	2.3 ± 0.3 *^c^*	0.20 ± 0.02 *^c^*	1.3 (1.4)	11 ± 1 *^e^*	6.1 ± 0.9 *^cd^*	7.4
D286H	2.7 ± 0.4 *^abc^*	6.1 ± 0.3 *^a^*	0.68 ± 0.1 *^a^*	2.3 (2.6)	23 ± 1 *^a^*	14 ± 2 *^a^*	11
D286I	2.4 ± 0.5 *^bc^*	3.8 ± 0.2 *^b^*	0.12 ± 0.03 *^c^*	1.6 (1.7)	17 ± 1 *^cd^*	5.6 ± 0.1 *^cd^*	20
D286M	1.8 ± 0.5 *^cd^*	4.2 ± 0.4 *^b^*	0.082 ± 0.01 *^c^*	2.3 (2.4)	20 ± 1 *^bc^*	4.8 ± 0.6 *^d^*	46
D286R	3.6 ± 0.5 *^a^*	5.9 ± 0.2 *^a^*	0.38 ± 0.08 *^b^*	1.6 (1.7)	22 ± 0.6 *^ab^*	7.0 ± 1 *^cd^*	16
D286V	3.2 ± 0.4 *^ab^*	4.1 ± 0.3 *^b^*	0.38 ± 0.03 *^b^*	1.3 (1.4)	16 ± 1 *^d^*	7.5 ± 1 *^c^*	14

## Supplementary Materials

The following are available online at https://www.mdpi.com/2309-608X/6/4/295/s1: Table S1: Primers used for site-directed mutagenesis. Figure S1: Multiple sequence alignment (MSA) of sequences of characterized GH29 α-l-fucosidases. Figure S2: LC–ESI–MS spectra and fragmentation patterns of fucosyllactose isomers. Figure S3: Reaction time courses for rationally designed variants. Figure S4: Homology models of representative D286 variants. Figure S5: Reaction time courses for D286 SSM variants. Figure S6: Size-exclusion chromatogram of xyloglucan substrate with and without xyloglucanase treatment. Figure S7: Reaction time courses for selected D286 variants with xyloglucanase-treated substrate.
