# Supplementary material for: Improved Transglycosylation by a Xyloglucan-Active α-l-Fucosidase from Fusarium graminearum
Source: J Fungi (Basel). 2020 Nov 18;6(4):295. doi: 10.3390/jof6040295 (PMC7711723; doi:10.3390/jof6040295)
Supplement: Supplementary file 1 [file jof-06-00295-s001.pdf]

Supplementary material for

Improved transglycosylation by a xyloglucan-active α-L-fucosidase from *Fusarium graminearum*

Birgitte Zeuner, Marlene Vuillemin, Jesper Holck, Jan Muschiol, Anne S. Meyer*
Department of Biotechnology and Biomedicine, Technical University of Denmark, 2800 Kgs. Lyngby, Denmark
*Corresponding author: asme@dtu.dk

Table S1. Primers used for site-directed mutagenesis. Mutated codons are underlined. Lowercase f indicated forward primers, while lowercase r indicates reverse primers.

Primer Name	Sequences
D286A_f	T ACT TCT GCT GTT GAT GCT TGG GAG AGA GGT GGT C
D286_r	ATC AAC AGC AGA AGT ATT TCT GAA ACC ATG ATC GTG
R289F_f	GTT GAT GAT TGG GAG TTT GGT GGT CCT TCT AAC TTG
R289_r	CTC CCA ATC ATC AAC AGC AGA AGT ATT TCT GAA AC
D286A-R289F_f	T ACT TCT GCT GTT GAT GCT TGG GAG TTT GGT GGT C
L340P_f	CT AAA AAC GGT AAC ATG CCT TTG AAC ATT TCT CCT ATG GC
L340_r	CAT GTT ACC GTT TTT AGA AAC TCT ATC CAA CAA AGA G
Y32I_f	GCA AAG TTC GGA GTT ATT TGG CAT TGG GGT GCT T
Y32_r	AAC TCC GAA CTT TGC GTC TCT ATA CCA TTC AGG
S228F_f	C ATC TGG AAC GAT TTC TTT TTG GAT TCT CCT GGT G
S228_r	GAA ATC GTT CCA GAT GAT ATC TGG TTG AAC GTG ATC
D286W_f	T ACT TCT GCT GTT GAT TGG TGG GAG AGA GGT GGT C
D286Y_f	T ACT TCT GCT GTT GAT TAC TGG GAG AGA GGT GGT C
D286G_f	T ACT TCT GCT GTT GAT GGT TGG GAG AGA GGT GGT C
D286I_f	T ACT TCT GCT GTT GAT ATT TGG GAG AGA GGT GGT C
D286L_f	T ACT TCT GCT GTT GAT TTG TGG GAG AGA GGT GGT C
D286P_f	T ACT TCT GCT GTT GAT CCT TGG GAG AGA GGT GGT C
D286V_f	T ACT TCT GCT GTT GAT GTT TGG GAG AGA GGT GGT C
D286F_f	T ACT TCT GCT GTT GAT TTT TGG GAG AGA GGT GGT C
D286E_f	T ACT TCT GCT GTT GAT GAA TGG GAG AGA GGT GGT C
D286R_f	T ACT TCT GCT GTT GAT AGA TGG GAG AGA GGT GGT C
D286H_f	T ACT TCT GCT GTT GAT CAC TGG GAG AGA GGT GGT C
D286K_f	T ACT TCT GCT GTT GAT AAA TGG GAG AGA GGT GGT C
D286S_f	T ACT TCT GCT GTT GAT TCT TGG GAG AGA GGT GGT C
D286T_f	T ACT TCT GCT GTT GAT ACT TGG GAG AGA GGT GGT C
D286C_f	T ACT TCT GCT GTT GAT TGT TGG GAG AGA GGT GGT C
D286M_f	T ACT TCT GCT GTT GAT ATG TGG GAG AGA GGT GGT C
D286N_f	T ACT TCT GCT GTT GAT AAT TGG GAG AGA GGT GGT C
D286Q_f	T ACT TCT GCT GTT GAT CAA TGG GAG AGA GGT GGT C
D286R-E288A_f	GCT GTT GAT AGA TGG GCT AGA GGT GGT CCT TC
D286R-E288A_r	CCA TCT ATC AAC AGC AGA AGT ATT TCT GAA ACC ATG
E234A_f	G GAT TCT CCT GGT GCT TGT GGT TCT TTC GAG GG
E234_r	ACC AGG AGA ATC CAA AGA GAA ATC GTT CCA GAT GAT ATC TG
D286M-E288A_f	GCT GTT GAT ATG TGG GCT AGA GGT GGT CCT TC
D286M-E288A_r	CCA CAT ATC AAC AGC AGA AGT ATT TCT GAA ACC ATG
E288A_f	GCT GTT GAT GAT TGG GCT AGA GGT GGT CCT TC
E288_r	CCA ATC ATC AAC AGC AGA AGT ATT TCT GAA ACC ATG

```
FgFCO1     32  ..........................YWHWGAFTTAQYA.....................
TmaFuc     32  ..........................FIHWGIYSVPGWA.....TPTGELGKVPMDAWFF
BT2970     64  ..........................FIHWGLYAIPGGE................WNGKV
TfFuc1     47  ..........................FIHWGIYSMLADG.....................
AlfB       19  ..........................FIHWGLYSQMAVG.....................
AlfC       16  ..........................MIHWGLYSLLAGE................YRGES
Mfuc6      16  ..........................FVHWGHCSQHGCE.....................
aLfuk1     21  ..........................FIHWGLYAIPARG.....................
Mfuc5      17  ..........................FIHWGIYAIPARG.....................
Mfuc2      23  ..........................FIHWGIYSAAARH.....................
Mfuc7      24  ..........................FIHWGLYAMPGRH.....................
Mfuc1      22  ..........................FIHWGLYALPSRH.....................
Mfuc4      22  ..........................FIHWGLYSLPSRH.....................
FoFCO1    175  ..........................FIHWGLYSVPAFG................NRPGP
NixE      111  ..........................FIHWGVFSVPAFG.....................
Blon0248   33  ..........................FAHWGSYSVPAWAEPI....GALGTFDDPVYWNT
Blon0426   33  ..........................FAHWGSYSVPAWAEPI....GALGTFDDPVYWNT
DdFuc      45  ..........................FIHFGIYSVPAFA.....................
DmFuca     58  ..........................FLHYGVYSVPSFG.....................
PapFuC     56  ..........................FMHWGVFSVPSFG.....................
RnFucA1    57  ..........................FVHWGVYSVPAWG.....................
ClFuc      61  ..........................FVHWGEFAVPAWG.....................
FucA1      56  ..........................FIHWGVFSVPAWG.....................
AlfA       26  ..........................IIHWGPYSVPAYAPVNIPDYGTLIKTKSLGYLFE
BT2192     47  ..........................FFHFGINTFE........................
BbAfcB    500  AALKTKLSKLRPTEGQADYIDWGQIGFLHYGINTYYNQ......................
AtFUC1     53  ..........................FLHFGPNTFTDS......................
Strep      94  ..........................FTHFGMNTFTGR......................
BT1625     66  ..........................FLHFGINTFTGR......................
BT4136     66  ..........................FLHFSINAFTGR......................
SpGH29     26  ..........................FIHFGPNTFYDQ......................
CpAfc2     63  ..........................FIHFGVNTFTGR......................
BiAfcB     34  ..........................FLHFGMNTMTDR......................

FgFCO1     45  ....SEW...YPRNMYEPDSD...QRKHHTETYGPPEEWGYENFIKGAKDKKGNFVQFKP
TmaFuc     61  QNPYAEW...YENSLRIKESP...TWEYHVKTYGENFE..YEKFA..............D
BT2970     82  YGGAAEW...LKSWAKVPADE...................WLKLM..............D
TfFuc1     60  .....EW...VMHNKNLNWEE...................YAKLA..............S
AlfB       32  .....EW...TELIHHRNQHD...................YEQLI..............K
AlfC       34  SSAYAEW...IQSKFQIPNAE...................YGNLA..............T
Mfuc6      29  ....LSWPL.VGGVAALPEAKRLSVAE.............YHSTA..............A
aLfuk1     34  .....EW...VRSFERIPVED...................YEKYF..............N
Mfuc5      30  .....EW...VKQQESIVESA...................YQPFF..............E
Mfuc2      36  .....EW...VKNLEKISDAD...................YQPYF..............D
Mfuc7      37  .....EW...VKSRERIPEER...................YDRYF..............R
Mfuc1      35  .....EW...VQSQEKIAPEV.Y.................YEKYF..............K
Mfuc4      35  .....EW...VQSQEQIPPET...................YRQYF..............E
FoFCO1    193  NQDYAEW...YGYRMTQPDFP.SQTYQYHRATYGENFN..YDDFV..............S
NixE      124  ....SEW...YSRNMYLEGSK...EFAHHVATYGPQASSGYKDLI..............P
Blon0248   63  HCPYAEW...YWNTMSIKGSP...AAEHQKEVYG.DMP..YEDFI..............D
Blon0426   63  HCPYAEW...YWNTMSIKGSP...AAEHQKEVYG.DMP..YEDFI..............D
DdFuc      58  NGGYAEW...YWWTLKNPSSDGGATQRYHEKEFGANFT..YQDFV..............S
DmFuca     71  ....SEWFWTNWKNLRNPEYV.QFMQRNYKPDFT......YQEFA..............S
PapFuC     69  ....SEWFWWHWQGKPLAPYV.EFMKKNYRPGFT......YPDFA..............P
RnFucA1    70  ....SEWFWWHWQGEQSSAYV.RFMKENYPPGFS......YADFA..............P
ClFuc      74  ....SEWFWWHWKGEGLPQYE.QFMSENYPPGFS......YADFG..............P
FucA1      69  ....SEWFWWHWQGEGRPQYQ.RFMRDNYPPGFS......YADFG..............P
AlfA       60  HQPYAEW...YGNSMLLPSSP...VAAYHRTHYG.NTS..YADFA..............K
BT2192     57  .....EY...VNEGDGKAST.......................................A
BbAfcB    538  .....EW....GHGNEDP.........................................S
AtFUC1     65  .....EW....GTGKANP.........................................S
Strep     106  .....EW....GSGTEDE.........................................K
BT1625     78  .....EW....GDGQEDP.........................................A
BT4136     78  .....EW....GDGKEDP.........................................N
SpGH29     38  .....EW....GTGQEDP.........................................E
CpAfc2     75  .....EW....GDGTENP.........................................E
BiAfcB     46  .....EW....GLGHEDP.........................................A
```

Figure S1. Continues on next page.

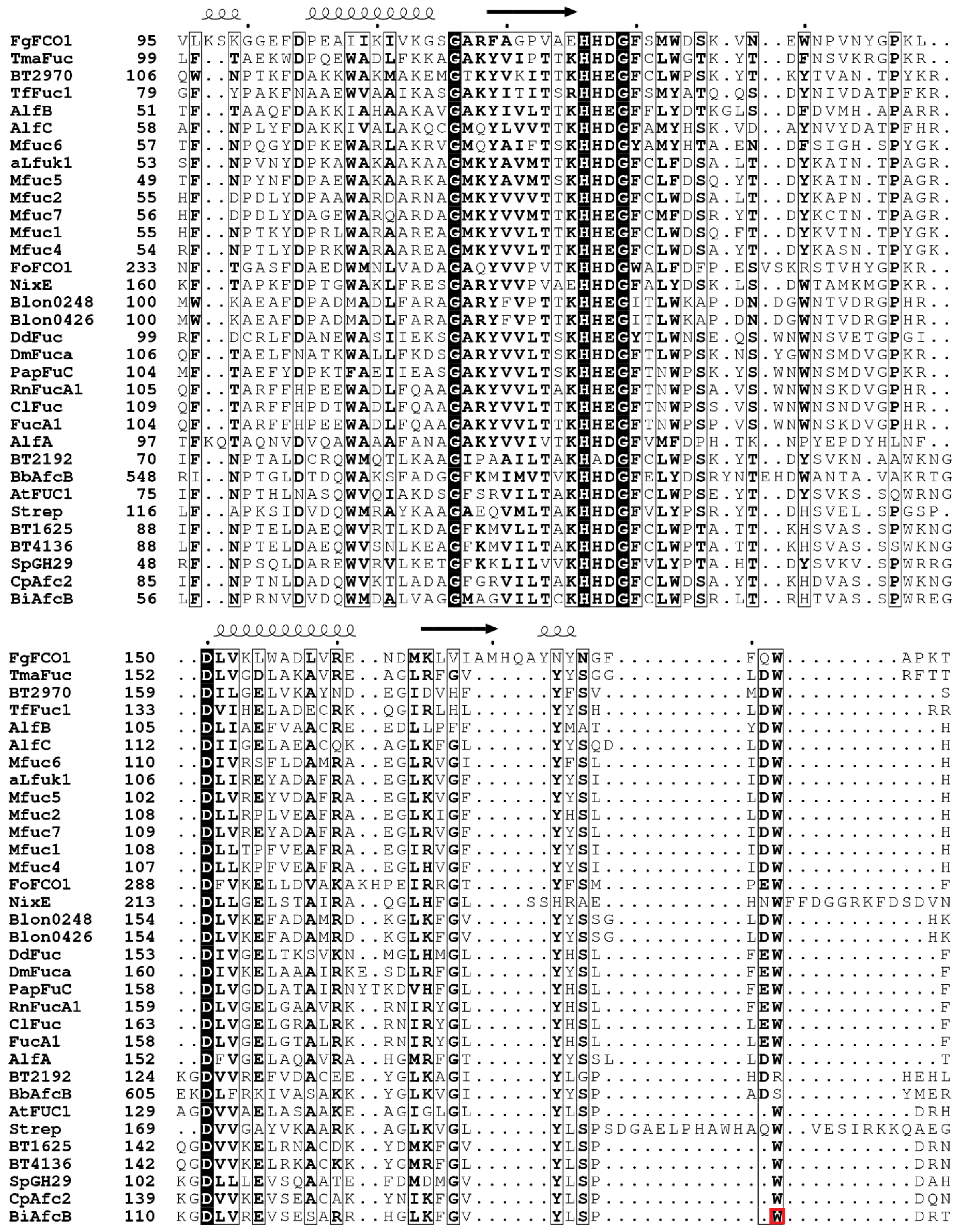

Figure S1. Continues on next page.

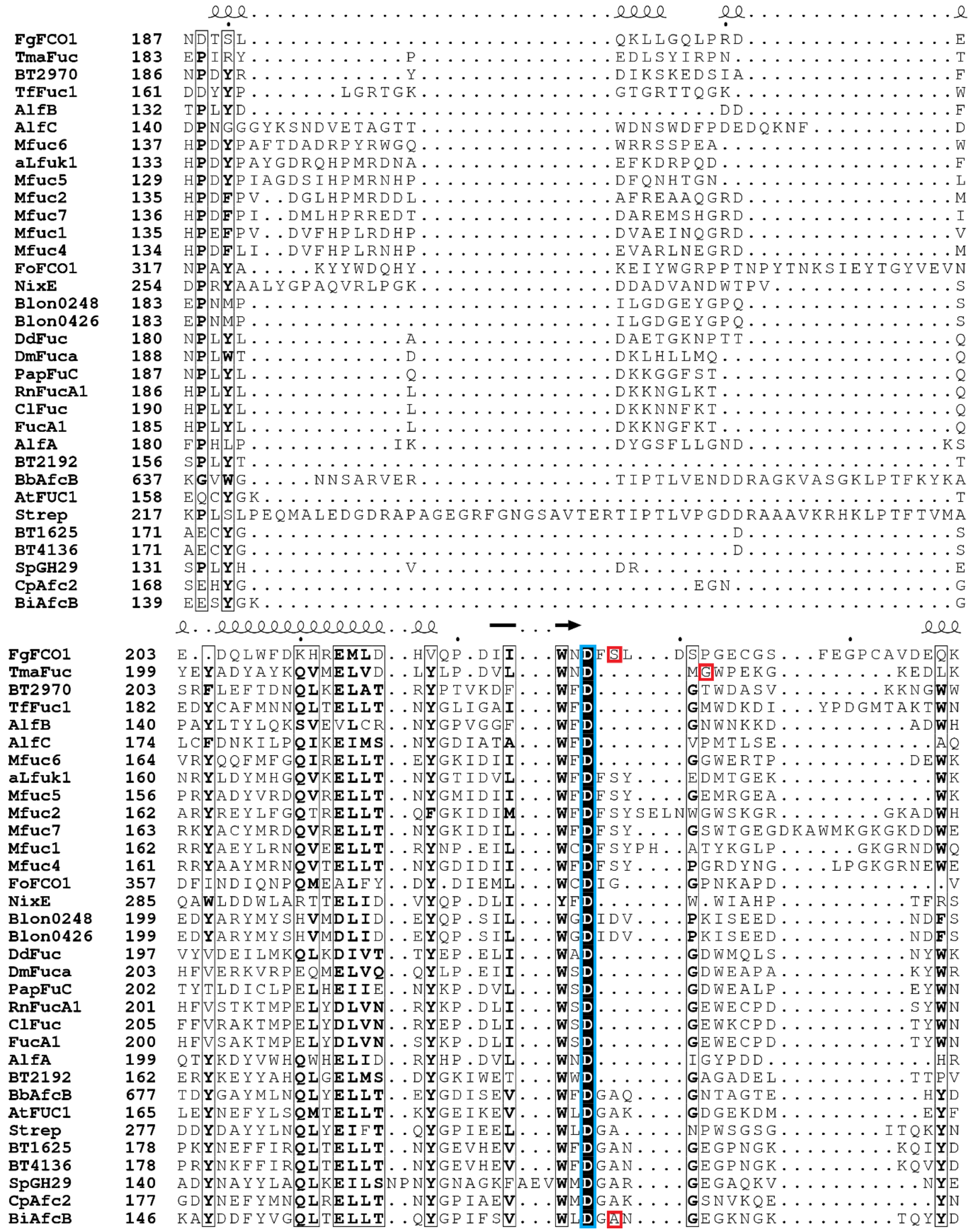

Figure S1. Continues on next page.

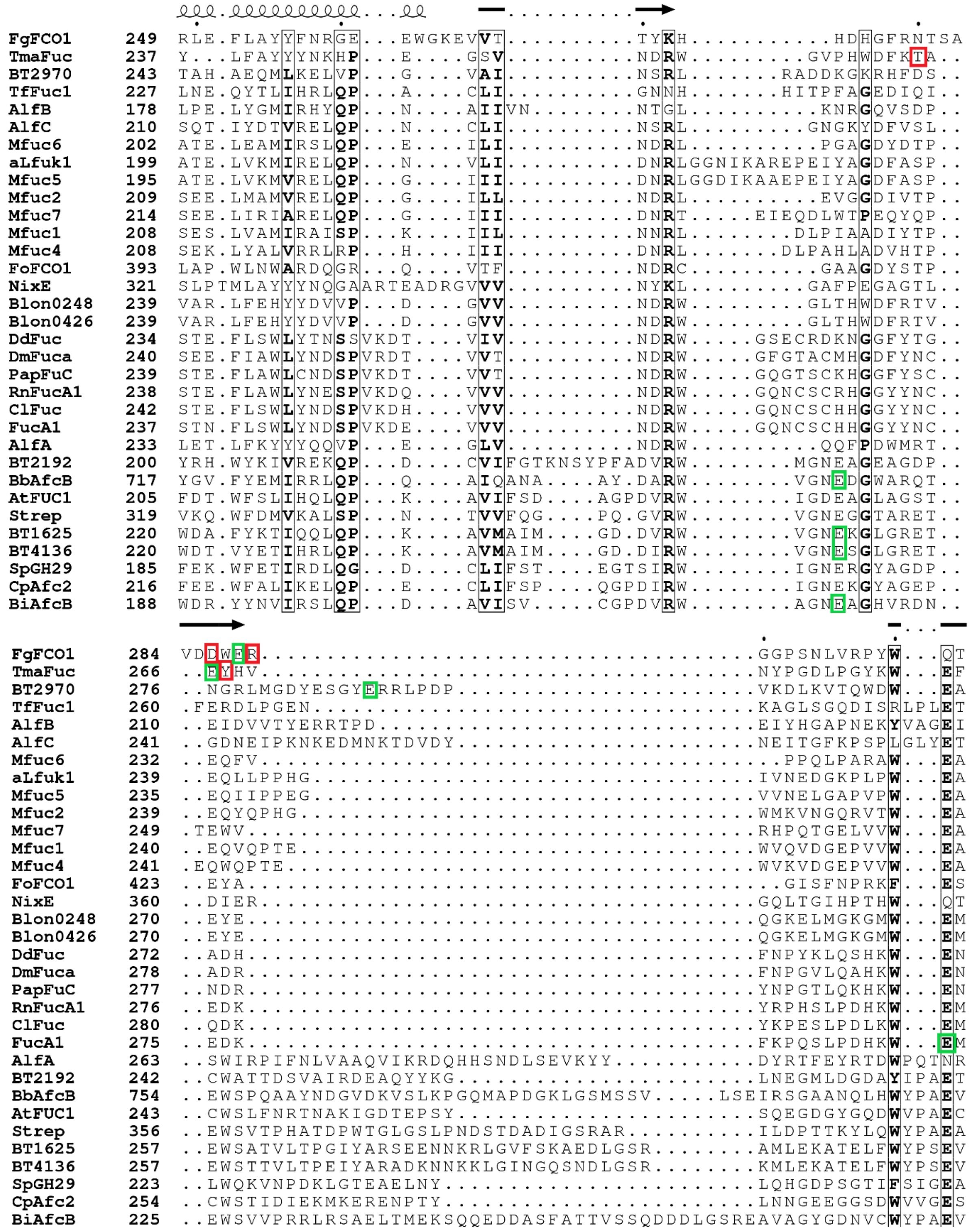

Figure S1. Continues on next page.

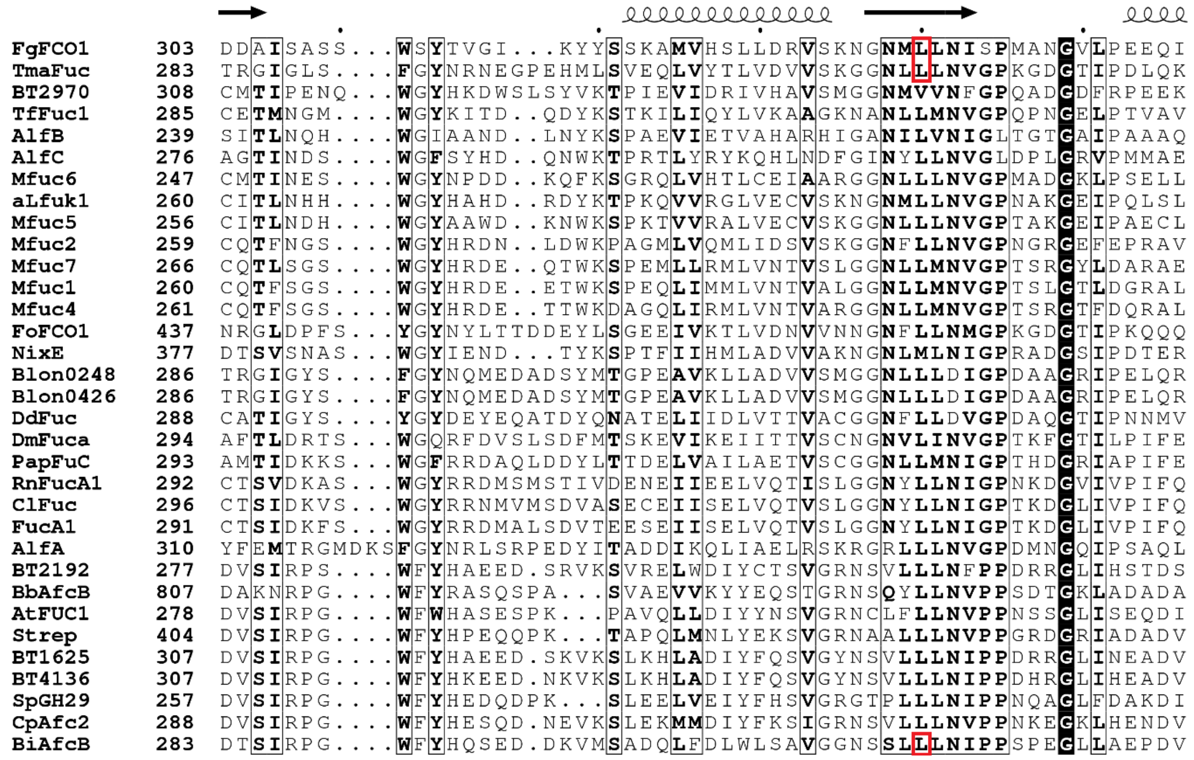

Figure S1. Multiple sequence alignment (MSA) of all bacterial and eukaryotic characterized GH29 sequences listed in CAZy January 2020. The alignment outside residues 32-356 of *Fg*FCO1 has been omitted for clarity. MSA performed with MUSCLE using default settings. The alignment was visualized by ESPript 3.0 [32] using PDB 4NI3 (*Fg*FCO1) as template for structural alignment. Numbers in the left column indicate residue numbering in each protein. Note that *Fg*FCO1 uses the numbering from the crystal structure [25] omitting the 24-amino acid signal peptide given in the GenBank sequence (AFR68935.1). Red boxes highlight residues mutated in the current work (*Fg*FCO1), or the best mutations identified in previous works (*Tm*αFuc [13] and *Bi*AfcB [6,34]). The blue box highlights the catalytic nucleophile, whereas the green boxes highlight the catalytic acid/base where known [25,36]. Entries listed from FgFCO1 to AlfA belong to GH29 subfamily A, whereas entries listed from BT2192 to BiAfcB belong to GH29 subfamily B. FgFCO1: *Fusarium graminearum* AFR68935.1; TmaFuc: *Thermotoga maritima* TAAD35394.1; BT2970: *Bacteroides thetaiotaomicron* AAO78076.1; TfFuc1: *Tannerella forsythia* AEW21393.1; AlfB: *Lactobacillus casei* CAQ67877.1; AlfC: *Lactobacillus casei* CAQ67984.1; Mfuc6: uncultured bacterium AIC77303.1; *Paenibacillus thiaminolyticus* CBM40947.1; Mfuc5: uncultured bacterium AIC77302.1; Mfuc2: uncultured bacterium AIC77299.1; Mfuc7: uncultured bacterium AIC77304.1; Mfuc1: uncultured bacterium AIC77298.1; Mfuc4: uncultured bacterium AIC77301.1; FoFCO1: *Fusarium oxysporum* AFR68934.1; NixE: *Xanthomonas campestris* pv. *campestris* AAM42160.1; Blon0248: *Bifidobacterium longum* subsp. *infantis* ACJ51376.1; Blon0426: *Bifidobacterium longum* subsp. *infantis* ACJ51546.1; DdFuc: *Dictyostelium discoideum* AAO51149.1; DmFuca: *Drosophila melanogaster* AAM50292.1; PapFuC: *Patiria pectinifera* BBG92283.1; RnFucA1: *Rattus norvegicus* CAA34268.1; ClFuc: *Canis lupus familiaris* CAA63362.1; FucA1: *Homo sapiens* AAA52481.1; AlfA: *Lactobacillus casei* CAQ67115.1; BT2192: *Bacteroides thetaiotaomicron* AAO77299.1; BbAfcB: *Bifidobacterium bifidum* BAH80310.1; AtFUC1: *Arabidopsis thaliana* NP_180377.2; Strep: AAD10477.1: *Streptomyces* sp.; BT1625: *Bacteroides thetaiotaomicron* AAO76732.1; BT4136: *Bacteroides thetaiotaomicron* AAO79241.1; SpGH29: *Streptococcus pneumoniae* AAK76203.1; CpAfc2: *Clostridium perfringens* ABG83106.1; BiAfcB: *Bifidobacterium longum* subsp. *infantis* ACJ53394.1.

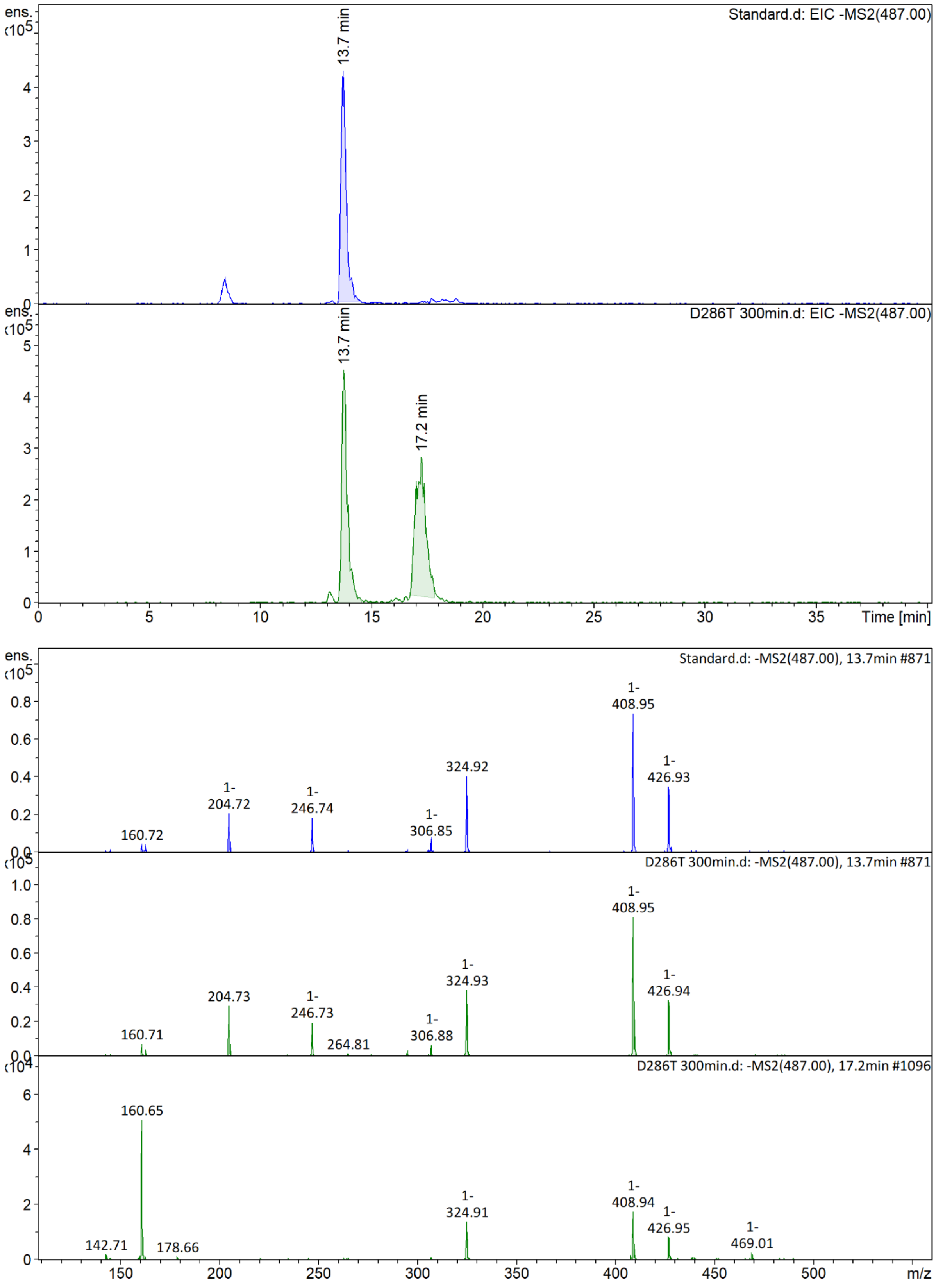

Figure S2. Top: Extracted ion chromatograms of selected ions (*m/z* 160.7, 204.7, 246.7, 307.0, 325.4, 427.0, 469.0) from MS^2 fragmentation of *m/z* 487 in samples containing 2'-fucosyllactose standard (blue) or the *Fg*FCO1 D286T reaction terminated after 300 minutes (green). Below: MS^2 fragmentation pattern of the mother ion *m/z* 487 corresponding to the peak at 13.7 minutes in the 2'-fucosyllactose standard or the two peaks at 13.7 and 17.2 minutes in the *Fg*FCO1 D286T reaction terminated after 300 minutes.

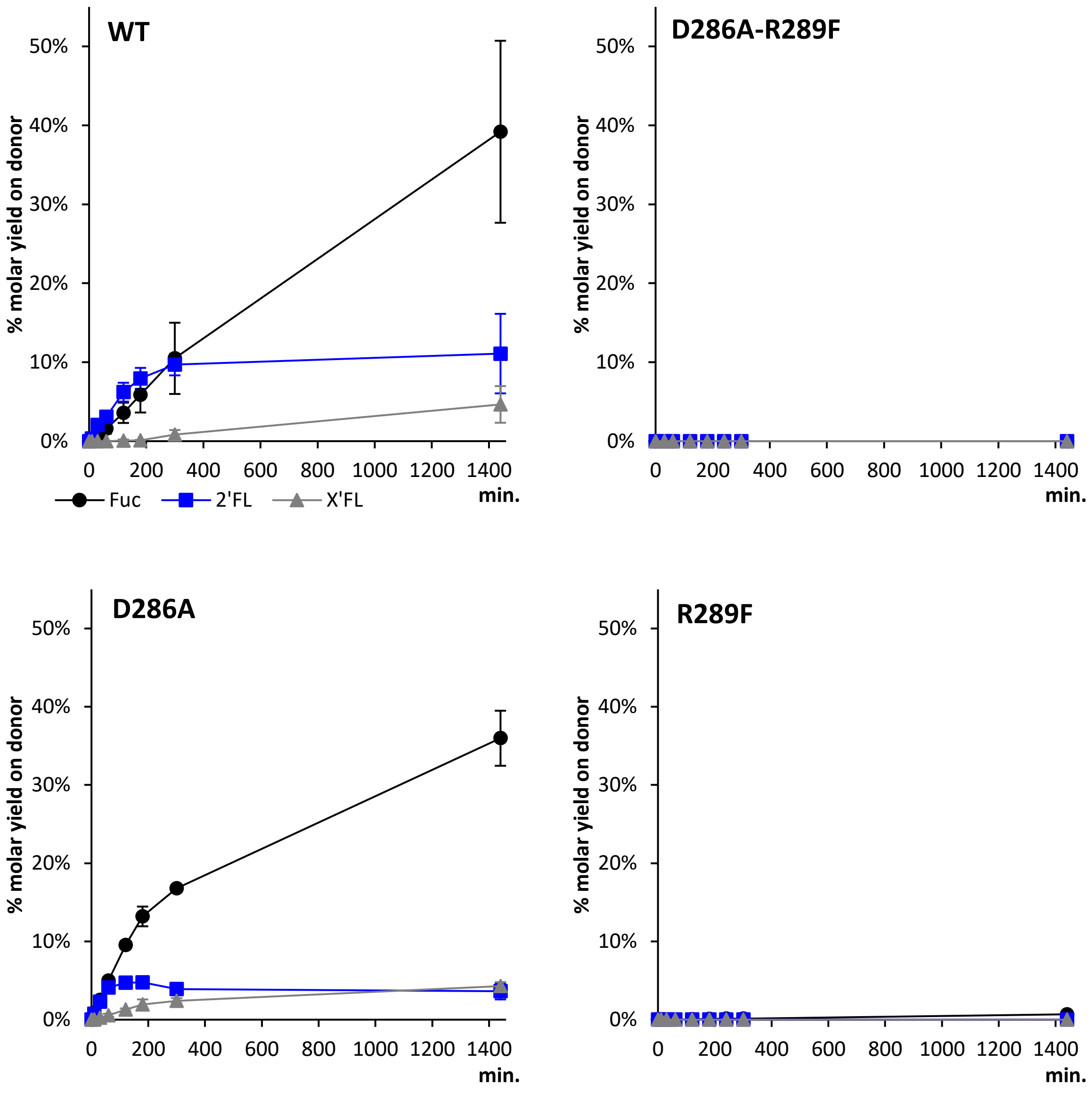

Figure S3. Molar yields on the donor substrate (2 mM Fuc bound in xyloglucan) of free fucose (Fuc; black circles), 2’-fucosyllactose (2’FL; blue squares), and the non-HMO fucosyllactose isomer X’FL (grey triangles) obtained with the WT and first rationally designed set of variants of *Fg*FCO1. The reaction was monitored for 24 h and took place with 100 mM lactose as acceptor substrate in 100 mM acetate buffer, pH 4.6, and 40°C using 2.5 µM of each enzyme variant. Error bars indicate standard deviation of two to four replicates for each data point.

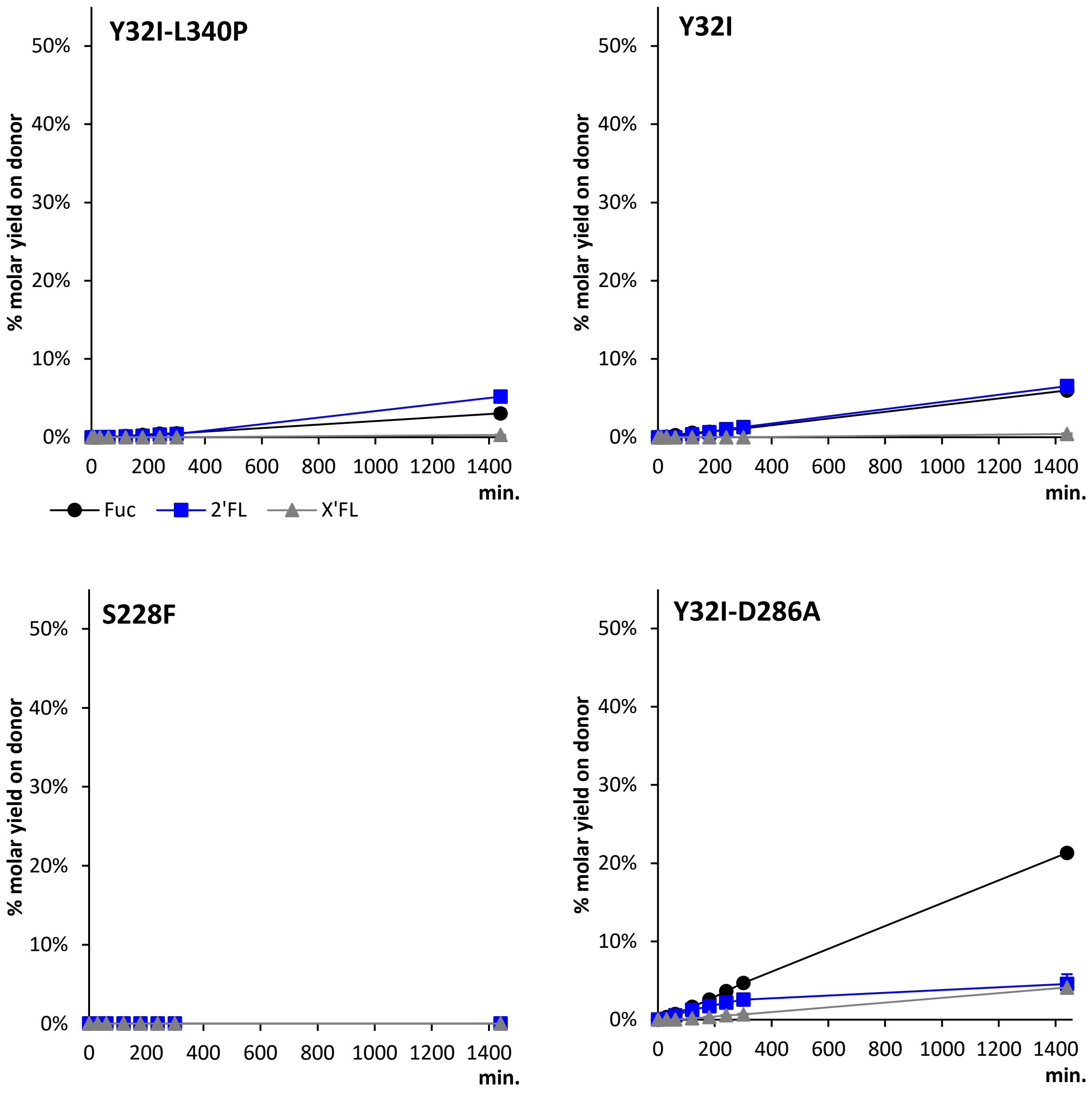

Figure S3 continued.

D286A aligned to WT:

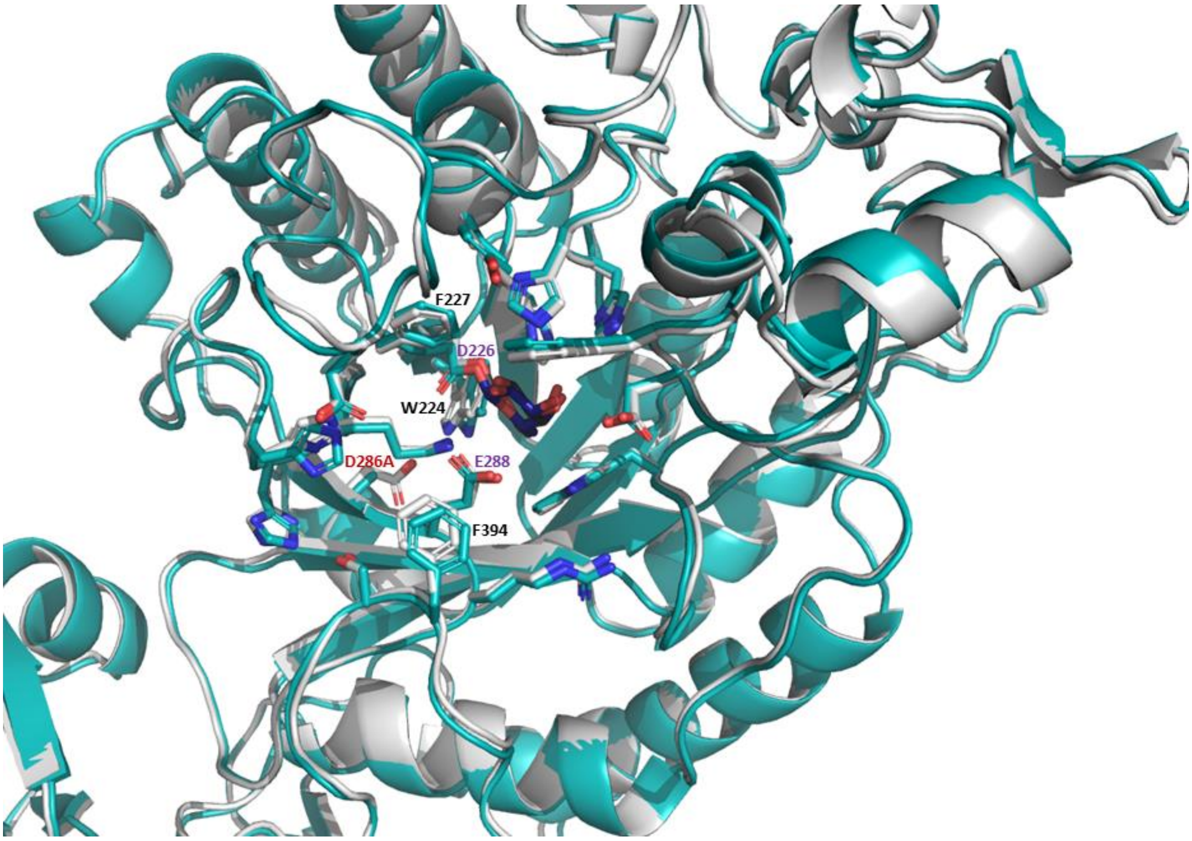

D286I aligned to WT:

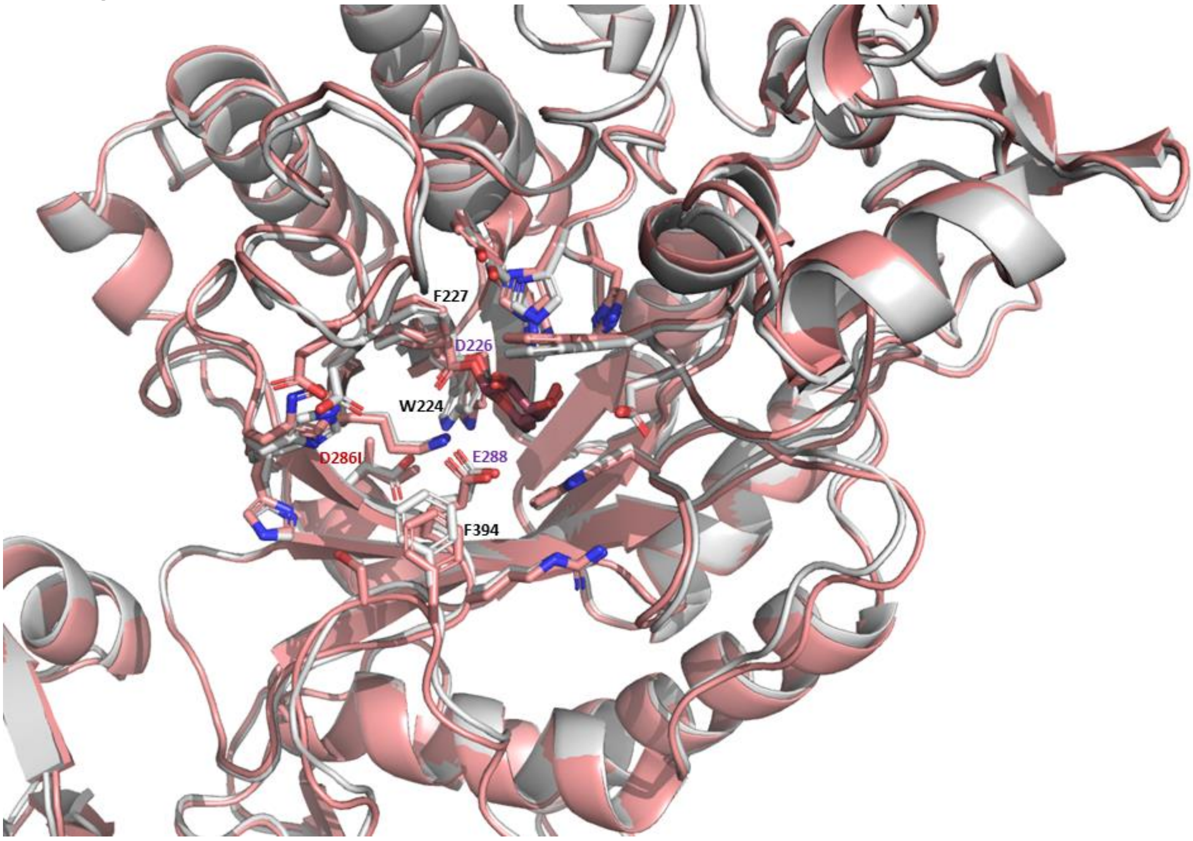

Figure S4. Homology models of representative D286 variants (coloured) aligned to the WT *Fg*FCO1 (grey). The Fuc ligand from PDB 4PSR (dark grey in WT, coloured in variants) is shown as a covalent intermediate with the catalytic nucleophile D226.

D286M aligned to WT:

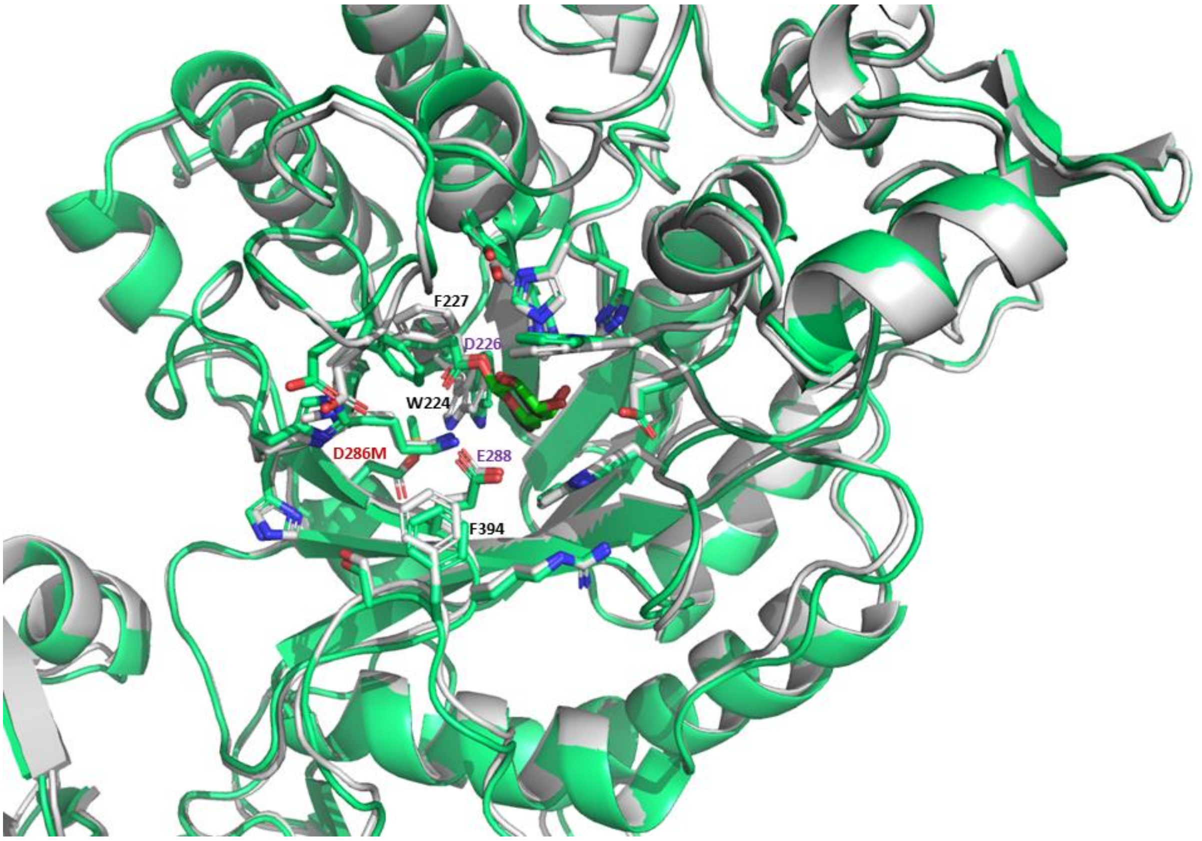

D286R aligned to WT:

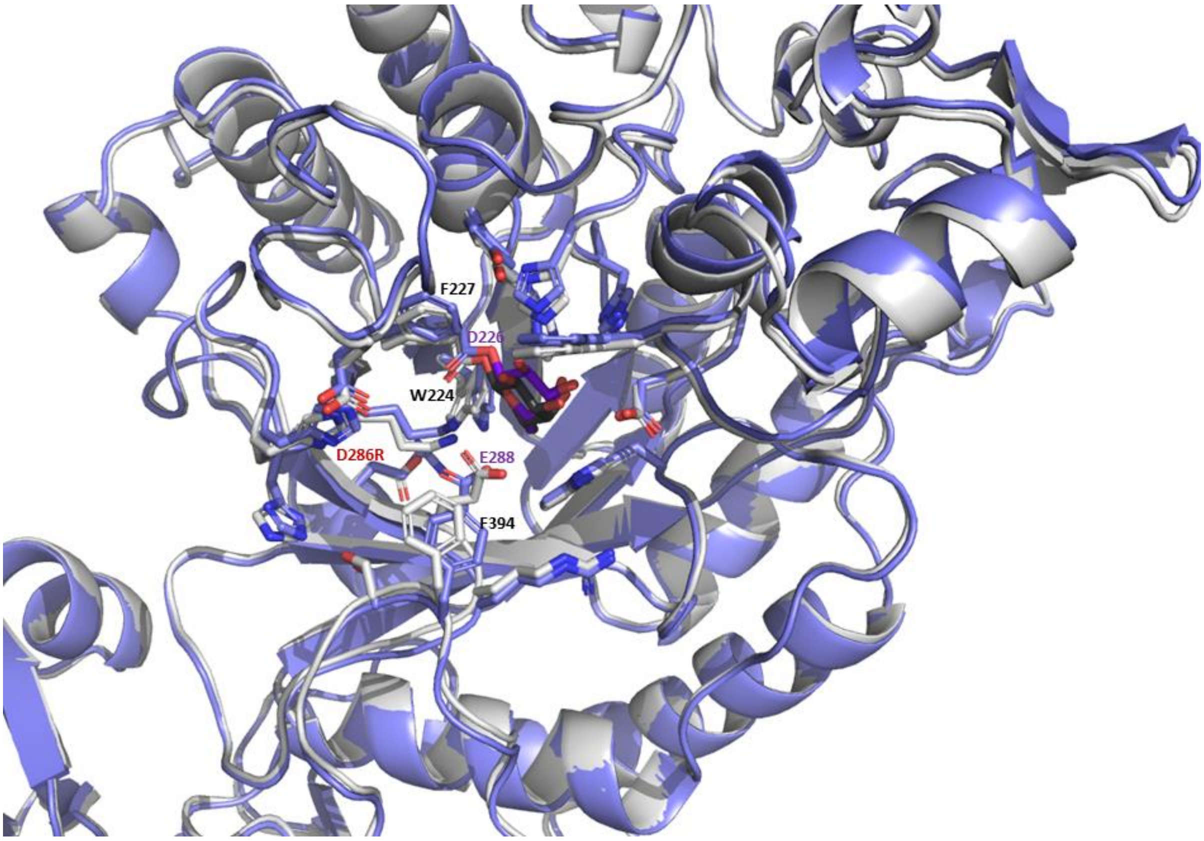

Figure S4. Continued.

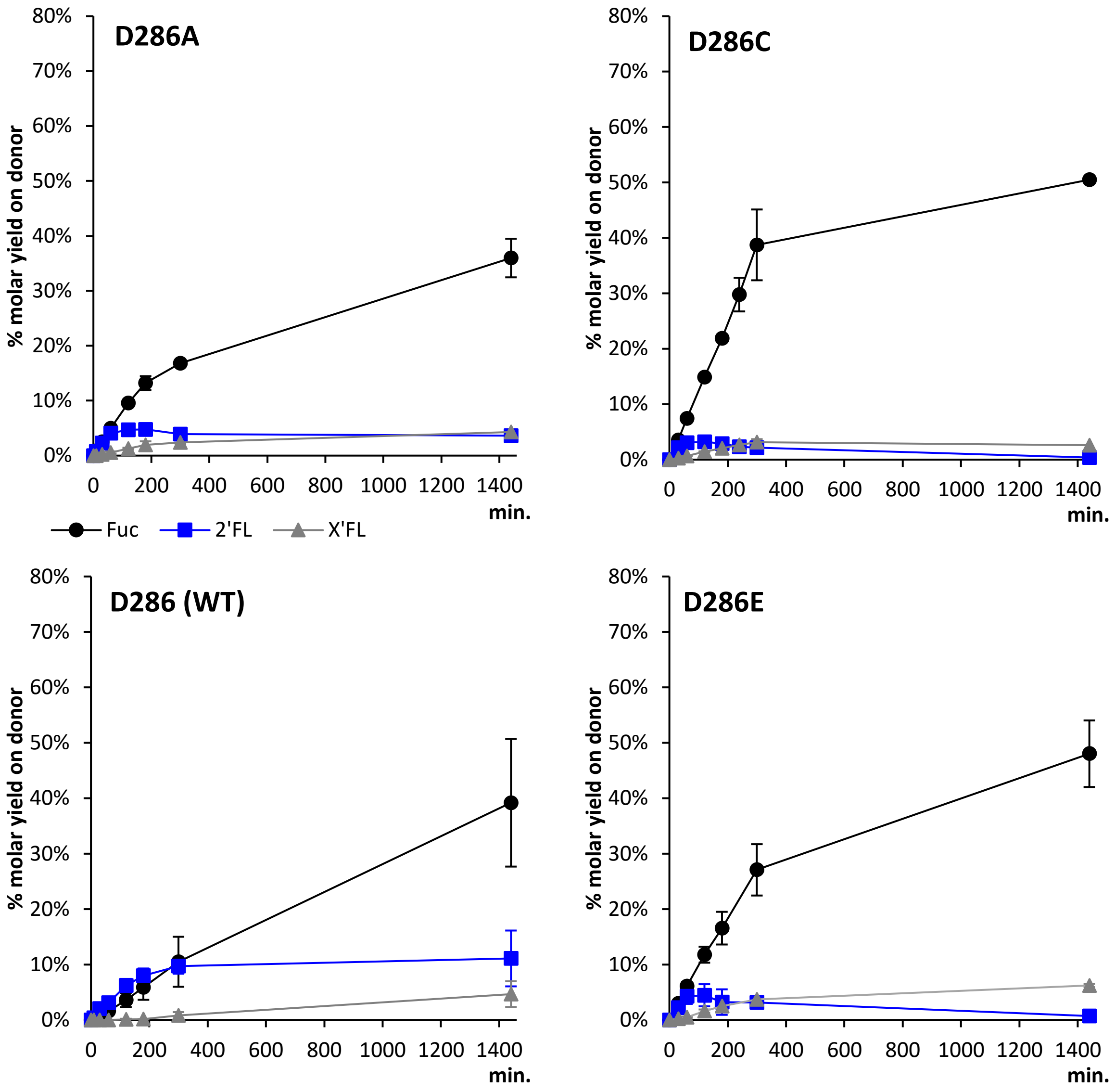

Figure S5. Molar yields on the donor substrate (2 mM Fuc bound in xyloglucan) of free fucose (Fuc; black circles), 2’-fucosyllactose (2’FL; blue squares), and the non-HMO fucosyllactose isomer X’FL (grey triangles) obtained with the site-saturation mutagenesis (SSM) variants of *Fg*FCO1 D286. The reaction was monitored for 24 h and took place with 100 mM lactose as acceptor substrate in 100 mM acetate buffer, pH 4.6, and 40°C using 2.5 µM of each enzyme variant. Error bars indicate standard deviation of two to four replicates for each data point.

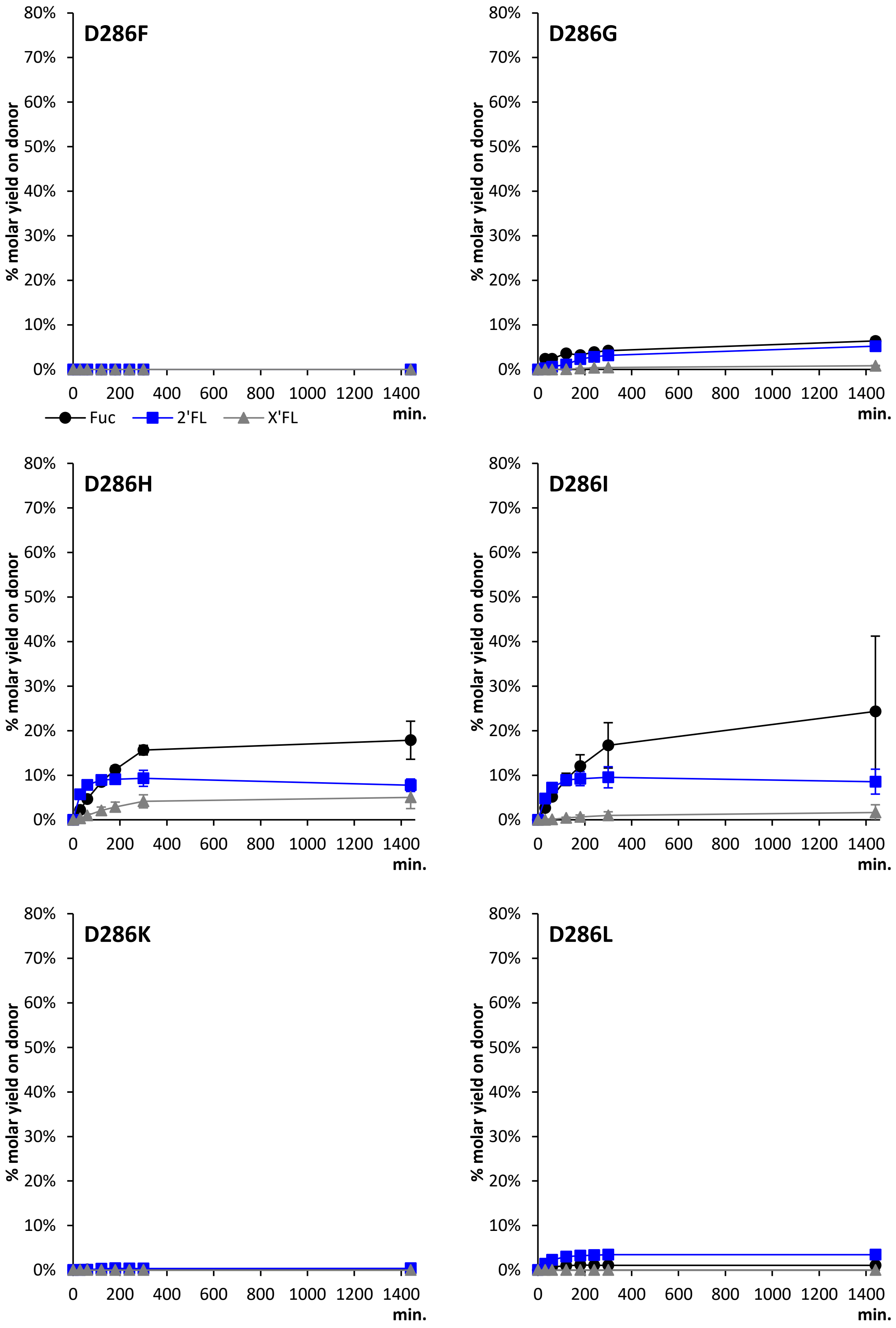

Figure S5 continued.

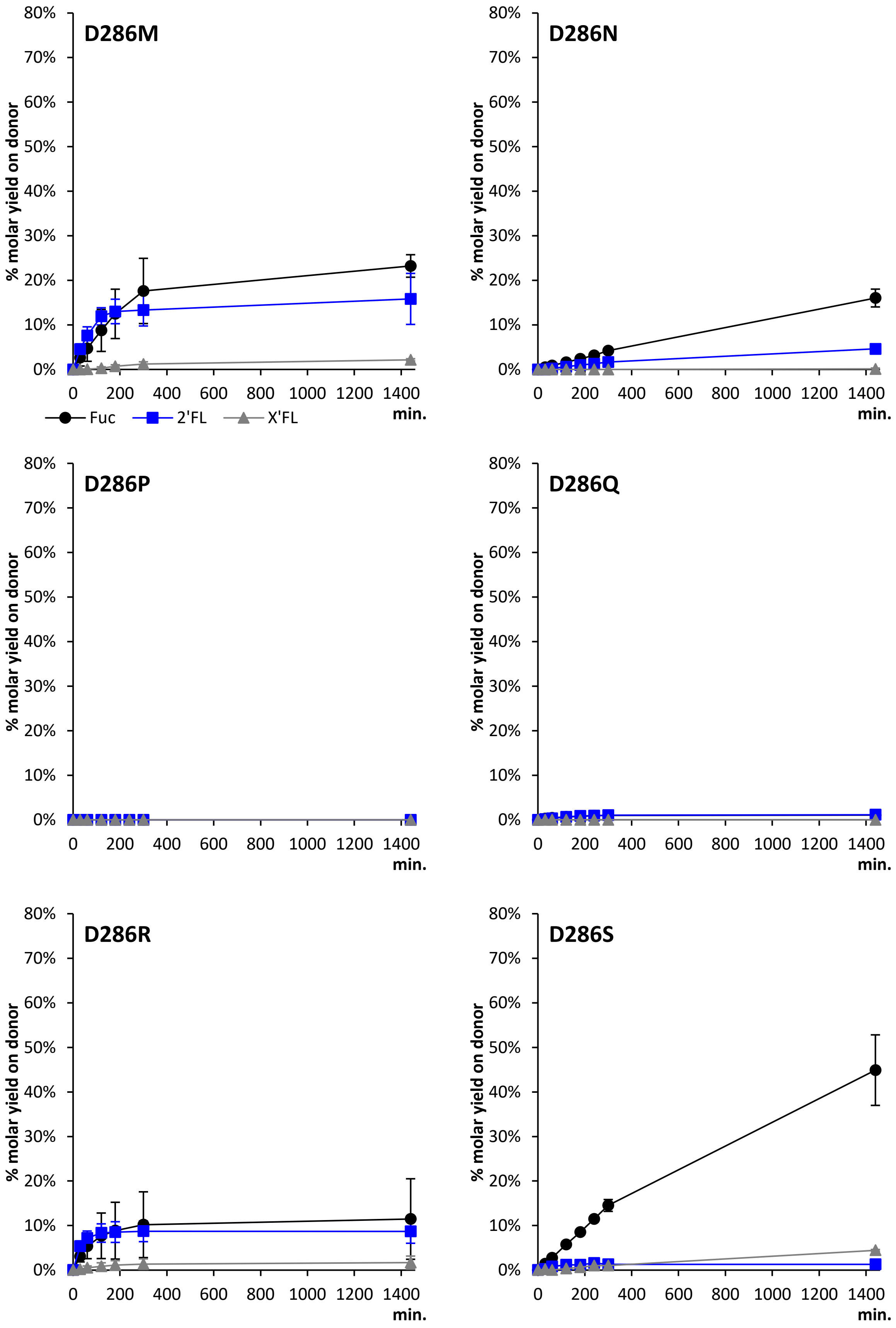

Figure S5 continued.

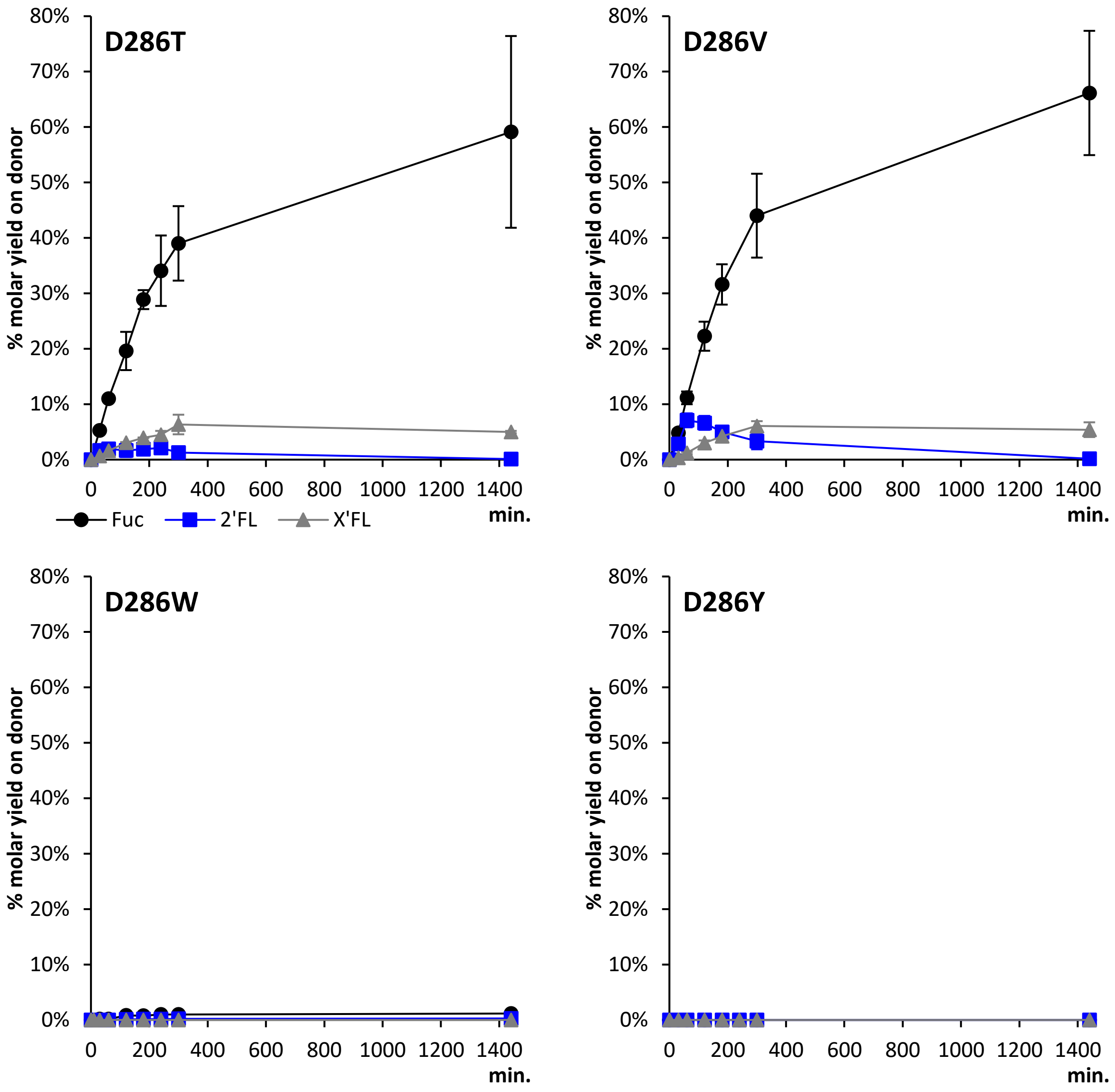

Figure S5 continued.

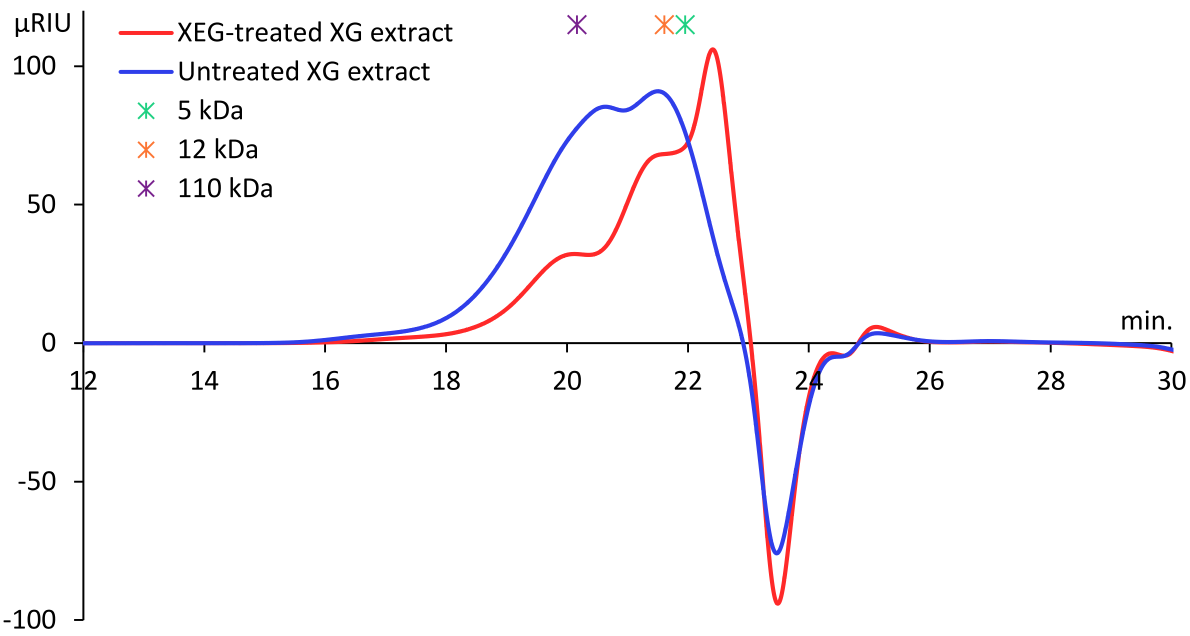

Figure S6. Size exclusion chromatogram (SEC) of the xyloglucan-rich fraction extracted from citrus peel at alkaline conditions and used as fucosyl donor substrate after treatment with 100 U/g of xyloglucan-specific endo-β-1,4-glucanase (XEG) for 1 h at 40°C followed by heating at 99°C for 10 minutes to inactivate the XEG (red). For comparison, untreated substrate subjected to the same heat treatment without XEG (blue). For size estimation, stars indicate retention times of the maximum peak intensity of pullulan standards: 5 kDa (bright green), 12 kDa (orange), 110 kDa (purple).

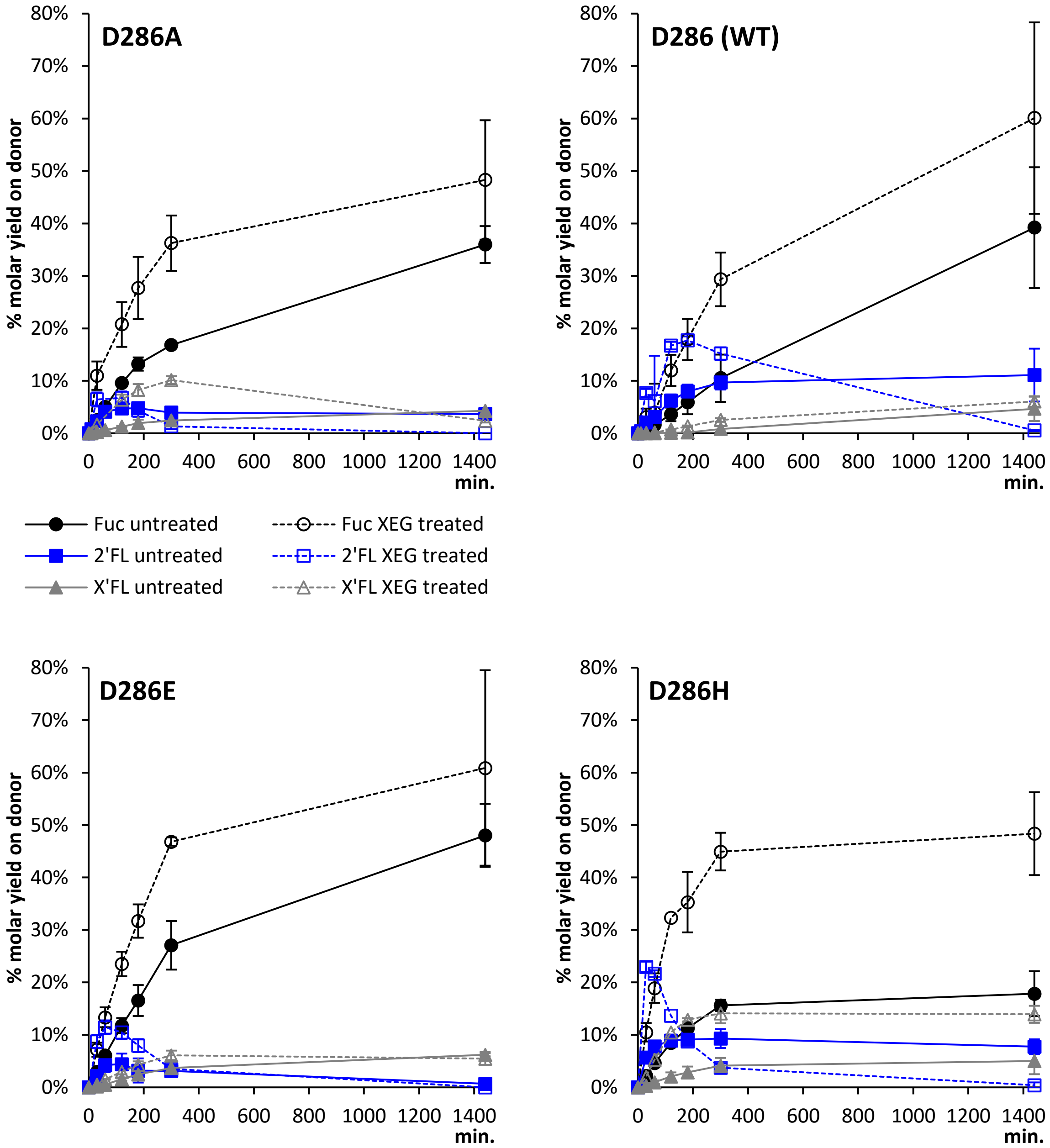

Figure S7. Molar yields on the donor substrate (2 mM Fuc bound in xyloglucan) of free fucose (Fuc; black circles), 2’-fucosyllactose (2’FL; blue squares), and the non-HMO fucosyllactose isomer X’FL (grey triangles) obtained with WT and variants of *Fg*FCO1 when using untreated citrus peel xyloglucan (solid lines and symbols) or xyloglucan-specific endo-β-1,4-glucanase (XEG) treated xyloglucan (dashed lines, open symbols) as donor substrates. The reaction was monitored for 24 h and took place with 100 mM lactose as acceptor substrate in 100 mM acetate buffer, pH 4.6, and 40°C using 2.5 µM of each enzyme variant. Error bars indicate standard deviation of two to four replicates for each data point.

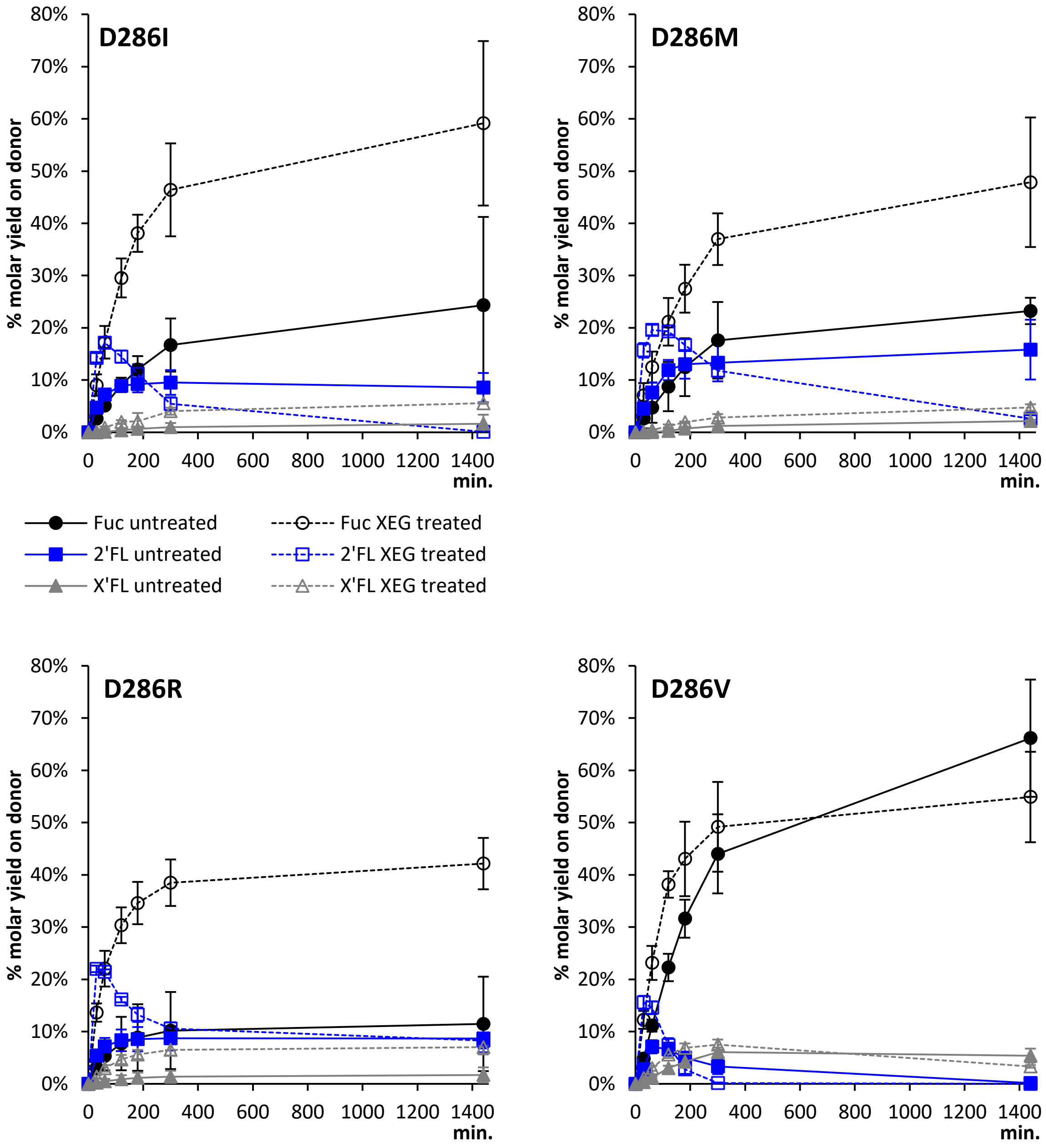

Figure S7 continued.
